# Identification of Phenylpyrazolone Dimers as a New Class of Anti‐*Trypanosoma cruzi* Agents

**DOI:** 10.1002/cmdc.201900370

**Published:** 2019-08-23

**Authors:** Maarten Sijm, Julianna Siciliano de Araújo, Alba Ramos Llorca, Kristina Orrling, Lydia Stiny, An Matheeussen, Louis Maes, Iwan J. P. de Esch, Maria de Nazaré Correia Soeiro, Geert Jan Sterk, Rob Leurs

**Affiliations:** ^1^ Division of Medicinal Chemistry Faculty of Sciences, Amsterdam Institute for Molecules, Medicines and Systems (AIMMS) Vrije Universiteit Amsterdam De Boelelaan 1108 1081 HZ Amsterdam The Netherlands; ^2^ Laboratório de Biologia Celular Oswaldo Cruz Institute (Fiocruz) Av. Brasil 4365, Manguinhos RJ 21040-900 Rio de Janeiro Brazil; ^3^ Laboratory for Microbiology, Parasitology and Hygiene (LMPH) Universiteit Antwerpen Universiteitsplein 1 2610 Antwerp Belgium

**Keywords:** benznidazole, Chagas disease, phenotypic screening, nifurtimox, *Trypanosoma cruzi*

## Abstract

Chagas disease is becoming a worldwide problem; it is currently estimated that over six million people are infected. The two drugs in current use, benznidazole and nifurtimox, require long treatment regimens, show limited efficacy in the chronic phase of infection, and are known to cause adverse effects. Phenotypic screening of an in‐house library led to the identification of 2,2′‐methylenebis(5‐(4‐bromophenyl)‐4,4‐dimethyl‐2,4‐dihydro‐3*H*‐pyrazol‐3‐one), a phenyldihydropyrazolone dimer, which shows an in vitro pIC_50_ value of 5.4 against *Trypanosoma cruzi*. Initial optimization was done by varying substituents of the phenyl ring, after which attempts were made to replace the phenyl ring. Finally, the linker between the dimer units was varied, ultimately leading to 2,2′‐methylenebis(5‐(3‐bromo‐4‐methoxyphenyl)‐4,4‐dimethyl‐2,4‐dihydro‐3*H*‐pyrazol‐3‐one (NPD‐0228) as the most potent analogue. NPD‐0228 has an in vitro pIC_50_ value of 6.4 against intracellular amastigotes of *T. cruzi* and no apparent toxicity against the human MRC‐5 cell line and murine cardiac cells.

## Introduction

Chagas disease, a neglected parasitic disease mainly endemic in Latin America, is caused by the kinetoplast *Trypanosoma cruzi*.^1^ This protozoan parasite can be transmitted by various routes, such as during the bloodmeal of infected insect vectors belonging to the triatomine family, through iatrogenic practices, mother‐to‐child transmission, laboratory accidents, and by oral ingestion of contaminated beverages.^2^ Over six million people are estimated to be infected, causing over 10 000 deaths each year.^3^ The impact of this disease is not only limited to Latin America, but has lately been spreading toward North America as well as other parts of the globe.^4^ While the insect vector is still only present in Latin America and certain parts of North America, the disease can enter blood banks via (labor) migrants or infected travelers.^5^ Chagas disease occurs in two distinct phases. During the acute phase, which lasts about six to eight weeks, there is positive parasitemia but the symptoms are generally mild and/or flu‐like. Therefore, this stage often passes without diagnosis of the disease.^6^ Due to the host immune response, the parasite load is controlled, and the majority of patients move to a chronic stage after two months, which is generally asymptomatic: i.e., the patient is infected and able to transmit, but without clear clinical pathological signs of the disease.^1^ However, after years or even decades, approximately 30 % of infected patients develop progressive chronic cardiomyopathy, while another 10 % develop digestive, neurological, or mixed clinical symptoms.^7^

**Figure 1:**
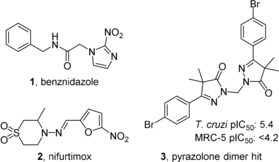
Current drugs against Chagas disease: the nitroimidazole benznidazole (**1**), the nitrofuran nifurtimox (**2**), and the identified phenyldihydropyrazolone dimer hit **3**.

Current first‐line treatments are the nitroaromatic compounds benznidazole (**1**) and nifurtimox (**2**) (Figure 1). Both drugs have proven their efficacy during the acute phase of the disease; however, efficacy during the chronic phase is much less evident.^8^, ^9^ In addition, both have adverse effects that lead to discontinuation of treatment, and natural resistance has also been noted.^10^, ^11^, ^12^, ^13^ Despite the need for novel anti‐*T. cruzi* chemotherapy, the drug discovery pipeline remains fairly empty. Herein we describe part of the research of the European Union (EU)‐funded, public–private consortium PDE4NPD that focused on identifying novel chemical candidates for neglected parasitic diseases with, amongst others, anti‐*T. cruzi* activity potential.

From the in vitro phenotypic screen of an in‐house compound library against our parasite panel, a phenyldihydropyrazolone dimer (**3**, Figure 1) was identified as a moderately potent antitrypanosomal agent (pIC_50_=5.4) against *T. cruzi* amastigotes, without toxicity toward human MRC‐5 cells (pIC_50_=<4.2).^14^ The in‐house library consisted of previously synthesized compounds in previous projects focused on antiparasitic drugs. Herein we report on the optimization of this new class of compounds as *T. cruzi* inhibitors.

### Chemistry

**Scheme 1:**
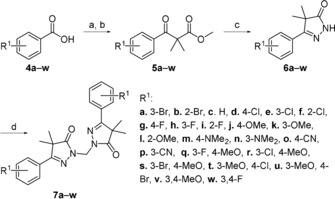
Reagents and conditions: a) (COCl)_2_, DMF, CH_2_Cl_2_, 2 h; b) methylisobutyrate, LDA, THF, −78 °C→RT, 18 h; c) N_2_H_4_⋅H_2_O, EtOH, 18 h, RT, 17–82 % (over two steps); d) TBAB, aq. NaOH, CH_2_Cl_2_, 60 °C, 18 h, 40–97 %.

The initial two steps of the synthetic route were adapted and modified from the work of Orrling et al.^15^ and were previously described by Sijm et al.^16^ The first step was the acid chloride formation of corresponding benzoic acids **4 a**–**w** (Scheme 1), which were subsequently condensed with the lithium enolate of methylisobutyrate. The formed keto esters **5 a**–**w** were condensed with hydrazine to yield the ring‐closed dihydropyrazolones **6 a**–**w**. These monomeric pyrazolones were then linked together with a methylene linker by holding at reflux in a biphasic system of CH_2_Cl_2_ and aqueous NaOH in the presence of tetra‐*n*‐butylammonium bromide (TBAB), yielding the desired pyrazolone dimers **7 a**–**w**.

**Scheme 2:**
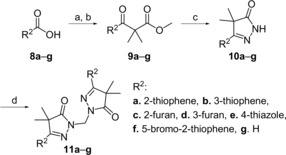
Reagents and conditions: a) (COCl)_2_, DMF, CH_2_Cl_2_, 2 h; b) methylisobutyrate, LDA, THF, −78 °C→RT, overnight; c) N_2_H_4_⋅H_2_O, EtOH, RT, 18 h, 16–64 % (over two steps); d) TBAB, aq. NaOH, CH_2_Cl_2_, 60 °C, 18 h, 38–69 %.

Synthesis of the heterocyclic substituted pyrazolones **11 a**–**g** (Scheme 2) was done using the same synthetic route as for the benzoic acids, although differences in solubility generally required different isolation methods. The synthesis of **11 g** was initiated with the goal of obtaining an oxazole‐substituted pyrazole. However, formation of the acid chloride proved to be extremely slow, and extra DMF had to be added. Subsequent addition of the lithium enolate of methylisoburate to the remaining Vilsmeier reagent was hypothesized to result in the formation of the unsubstituted pyrazolone.

**Scheme 3:**
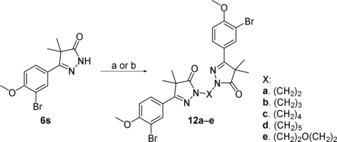
Reagents and conditions: a) alkyldihalide, NaH, DMF, 18 h, 39–84 %; b) 2‐bromoethyl ether, KOH, dioxane, 110 °C, 18 h, microwave, 45 %.

Installing the aliphatic linkers of two to five methylene units on the 3‐bromo‐4‐methoxypyrazolone **6 s** (Scheme 3) was done by deprotonation of the monomeric dihydropyrazolone unit followed by addition of the corresponding dihalide, yielding analogues **12 a**–**d**. The ethyl ether analogue **12 e** was synthesized by microwave reaction of the monomeric unit in the presence of 2‐bromoethyl ether and KOH.

**Scheme 4:**
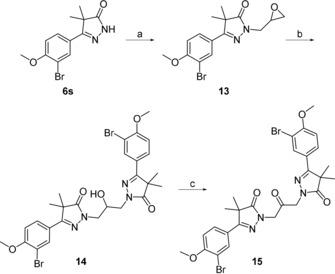
Reagents and conditions: a) epichlorohydrin, TBAB, aq. NaOH, 60 °C, 18 h, 52 %; b) **6 s**, K_2_CO_3_, DMF, 100 °C, 18 h, 53 %; c) DMP, CH_2_Cl_2_, RT, 2 h, 40 %.

Introduction of a 2‐propanol linker was done using a biphasic mixture of epichlorohydrin and aqueous NaOH, yielding epoxide‐substituted dihydropyrazolone **13** (Scheme 4). Reaction of this epoxide with a second monomeric unit in the presence of K_2_CO_3_ yielded the desired 2‐propanol‐linked dimer **14**. Oxidation of this dimer with Dess–Martin periodane (DMP) yielded the closely analogous 2‐propanone linked dihydropyrazolone **15**.

### Phenotypic screening

All compounds were initially tested for their in vitro antitrypanosomal activity against intracellular forms of *T. cruzi* (Tulahuen CL2, β‐galactosidase, a drug‐sensitive strain belonging to the discrete typing units, DTU VI), *T. brucei*, and *Leishmania infantum*, as well as for cytotoxicity on MRC‐5_SV2_ cells (human lung fibroblasts) and on primary mouse cardiac cell cultures.^17^ The full dataset is presented in Supporting Information Table 1. In‐depth profiling with selected compounds was performed using intracellular amastigotes from the Tulahuen strain transfected with β‐galactosidase and from the Y‐strain (DTU II). Additionally, bloodstream trypomastigotes of the Y‐strain were obtained from infected Swiss Webster donor mice.^16^

## Results and Discussion

With the discovery of the 4‐bromophenylpyrazolone dimer **3** (Figure 1) as a phenotypic hit against *T. cruzi*, SAR studies were initially focused on substituents of the phenyl ring, leading to compounds **7 a**–**z** (Table 1). Shifting the bromine to the 3‐ or 2‐position (**7 a**,**b**) resulted in two inactive compounds (pIC_50_ <4.2), whereas the unsubstituted phenyl analogue **7 c** was also inactive. To decrease the molecular weight, the bromine atom was replaced with other halogen substituents. The analogue with a chlorine atom at the 4‐position (**7 d**) shows similar activity as the 4‐bromophenyl hit, yet **7 d** is equally toxic against MRC‐5 cells. As observed with the bromine analogues **7 a**,**b**, the 2‐ and 3‐chloro analogues **7 e**,**f** are inactive. The 2‐ and 4‐fluoro analogues **7 g**,**i** are also inactive, while the 3‐fluorine analogue **7 h** has a pIC_50_ value of 5.2. Both the 3‐ and 4‐methoxyphenyl analogues **7 j**,**k** showed pIC_50_ values around 5.0, while the 2‐methoxy analogue **7 l** is inactive . The 3‐ and 4‐dimethylamino and cyano derivatives **7 m**–**p** are all inactive.

**Table 1:** In vitro activity of pyrazolone dimers against intracellular amastigotes of *T. cruzi* (Tulahuen strain) and on MRC‐5 cell growth by phenyldihydropyrazolone dimers with modifications on the phenyl ring.

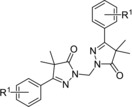					
Compd	R^1^			pIC_50_^[a]^	
	4‐	3‐	2‐	*T. cruzi*	MRC‐5
**3**	Br	H	H	5.4	<4.2
**7 a**	H	Br	H	<4.2	<4.2
**7 b**	H	H	Br	<4.2	<4.2
**7 c**	H	H	H	<4.2	<4.2
**7 d**	Cl	H	H	5.3	5.1
**7 e**	H	Cl	H	<4.2	<4.2
**7 f**	H	H	Cl	<4.2	<4.2
**7 g**	F	H	H	<4.2	<4.2
**7 h**	H	F	H	5.2	4.4
**7 i**	H	H	F	4.3	<4.2
**7 j**	OMe	H	H	5.0	<4.2
**7 k**	H	OMe	H	5.1	4.4
**7 l**	H	H	OMe	<4.2	<4.2
**7 m**	NMe_2_	H	H	<4.2	<4.2
**7 n**	H	NMe_2_	H	<4.2	<4.2
**7 o**	CN	H	H	<4.2	<4.2
**7 p**	H	CN	H	<4.2	<4.2
**7 q**	OMe	F	H	5.2	<4.2
**7 r**	OMe	Cl	H	5.9	<4.2
**7 s**	OMe	Br	H	6.4	<4.2
**7 t**	Cl	OMe	H	<4.2	<4.2
**7 u**	Br	OMe	H	<4.2	<4.2
**7 v**	OMe	OMe	H	4.3	<4.2
**7 w**	F	F	H	5.1	<4.2

[a] All reported values are within a standard deviation of ±0.2.

Combining halogen and methoxy substituents at the 3‐ and 4‐ positions of the phenyl ring was more successful. The 3‐fluoro‐4‐methoxyphenyl analogue **7 q** (Table 1) shows a pIC_50_ value of 5.2, while the 3‐chloro‐4‐methoxyphenyl dimer **7 r** shows increased activity, with pIC_50_=5.9. The most active molecule in this series is the 3‐bromo‐4‐methoxyphenyl **7 s** (NPD‐0228) which has a pIC_50_ value of 6.4, with no apparent cytotoxicity toward MRC‐5 cells. Switching the position of both substituents, as with compound **7 t** and **7 u**, resulted in totally inactive compounds (pIC_50_<4.2), which was surprising as the 4‐chloro‐ and 4‐bromo‐substituted phenyl analogues **3** and **7 c** performed better than their 3‐substituted counterparts **7 a** and **7 d**. Finally, the 3,4‐dimethoxyphenyl (**7 v**) and 3,4‐difluorophenyl (**7 w**) analogues showed only weak anti‐*T. cruzi* activity (pIC_50_: 4.3 and 5.1, respectively).

While the SAR study on the phenyl moiety yielded an improved hit **7 s** (Table 1, pIC_50_=6.4), its druglike properties are not ideal: molecular weight of 606 Da and a cLog*P* of 6.1. To optimize these parameters, several heterocycles were installed instead of the phenyl moiety. The 2‐ and 3‐thiophenes **11 a**,**b**, the 2‐ and 3‐furans **11 c**,**d**, and the 4‐thiazole **11 e** were unfortunately all inactive, with pIC_50_ values <4.2 (Table 2). As the original hit **3** contained a bromine substituent, this moiety was included on 2‐thiophene **11 f**, and some moderate activity (pIC_50_=5.1) was observed for this compound. In an attempt to introduce an oxazole substituent, the unsubstituted dihydropyrazolone **11 g**, obtained via ring‐closure of the intermediate Vilsmeier reagent, was tested, but also proved inactive.

**Table 2:** In vitro activity of pyrazolone dimers against intracellular amastigotes of *T*. *cruzi* (Tulahuen strain) and on MRC‐5 cells by dihydropyrazolone dimers with modification of the aromatic ring.

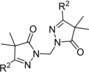			
Compd	R^2^	pIC_50_^[a]^	
		*T. cruzi*	MRC‐5
**11 a**	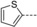	<4.2	<4.2
**11 b**	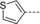	<4.2	<4.2
**11 c**	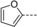	<4.2	<4.2
**11 d**	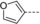	<4.2	<4.2
**11 e**	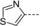	<4.2	<4.2
**11 f**	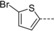	5.1	<4.2
**11 g**	H	<4.2	<4.2

[a] All reported values are within a standard deviation of ±0.2.

As the activities of the heterocyclic‐substituted pyrazolones were low, we decided to continue further investigations with the 4‐methoxy‐3‐bromophenyl‐substituted dihydropyrazolone **7 s**. Installing several aliphatic linkers with two to five methylene units (compounds **12 a**–**d**, Table 3) showed that the analogues with linkers of 2–4 carbon atoms (**12 a**–**c**) are all inactive, with pIC_50_ values below 4.3. Instead, the five‐carbon linker analogue **12 d** shows quite good activity with a pIC_50_ of 5.7. The other five‐atom linker, diethyl ether **12 e**, shows a pIC_50_ just above 5. However, this compound is equally toxic against MRC‐5 cells. While the 2‐propanol linker analogue **14** has a similar pIC_50_ as the five‐carbon linked analogue (5.7), it also showed quite high cytotoxicity (pIC_50_=5.2). The closely related ketone analogue **15** is totally inactive (Table 3).

**Table 3:** In vitro activity of pyrazolone dimers against intracellular amastigotes of *T. cruzi* (Tulahuen strain) and on MRC‐5 cell growth by dihydropyrazolone dimers with modification of the linker.

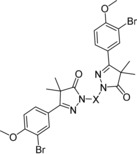			
Compd	X	pIC_50_^[a]^	
		*T*. *cruzi*	MRC‐5
**7 q**	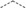	6.4	<4.2
**12 a**	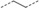	<4.2	<4.2
**12 b**	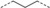	4.3	<4.2
**12 c**	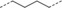	<4.2	<4.2
**12 d**	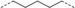	5.7	4.3
**12 e**	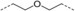	5.1	5.2
**14**	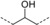	5.7	5.2
**15**	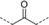	<4.2	<4.2

[a] All reported values are within a standard deviation of ±0.2.

Overall, optimization of the substituents on the phenyl ring gave valuable SAR data, and most importantly, the 3‐bromo‐4‐methoxy analogue **7 s** (Table 1) was confirmed as the most promising hit with sub‐micromolar potency (pIC_50_=6.4) against *T*. *cruzi* and over 100‐fold selectivity against MRC‐5 cells. Introduction of aromatic heterocycles on the dihydropyrazolone dimer did not result in potent compounds; only the 2‐bromothiophene **11 f** (Table 2) showed some activity (pIC_50_ 5.1). Finally, installing linkers with various lengths showed that the dimer with a five‐carbon linker (**12 d**, Table 3) shows a pIC_50_ of 5.7. All compounds listed in Tables 1–3 were screened against all parasites in a parasite panel (*T*. *brucei*, *T*. *cruzi*, and *L*. *infantum*); these data can be found in Supporting Information Table 1.

Next, the optimized hit **7 s** was screened in different assays against the intracellular amastigote form of *T*. *cruzi* (Tulahuen and Y‐strains) as well as the bloodstream form of the Y‐strain (Table 4). In both intracellular amastigote assays, NPD‐0228 (**7 s**) shows sub‐micromolar potencies with pIC_50_ values of 6.8 and 7.0, respectively. Additionally, measured pIC_90_ values are low micromolar (Table 4), while selectivity over mouse cardiac cells is >450‐fold. The hit **7 s** did not show any activity against the bloodstream trypomastigotes. In the same experiments, benznidazole (**1**, Figure 1) shows pIC_50_ values of 5.5 against the intracellular form and 4.9 against the bloodstream form of the Y‐strain.

**Table 4:** Activity and selectivity index (SI) of NPD‐0228 (**7 s**) against various *T. cruzi* strains.

Strain (form) of *T*. *cruzi*	pIC_50_^[a]^	pIC_90_^[a]^	SI^[b]^
Tulahuen (intracellular amastigotes)	6.8	6.2	>599
Y (intracellular amastigotes)	7.0	5.6	>455
Y (bloodstream trypomastigotes)	<4.3	ND	ND

[a] All reported values are within a standard deviation of ±0.2; ND: not determined. [b] Selectivity index was calculated against the pIC_50_ of mouse cardiac cells.

To investigate if the difference in activity against intracellular forms and bloodstream trypomastigotes is due to influence from the mammalian host cell triggering a mechanism to control and clear the parasite intracellularly, L929 cells were pretreated for 24 h with **7 s** at 0.17 μm (corresponding to the IC_50_ value on intracellular forms of the Tulahuen strain). Next, the cultures were washed to remove compound **7 s** and subsequently monitored for parasite infection. Our data show that drug administration only prior to the in vitro infection does not control the parasite load, leading to a maximum parasite reduction of 8.8 %, suggesting that the higher efficacy of NPD‐0228 (**7 s**) against the intracellular forms is due to activity against the intracellular parasite itself and is not related to an improved microbicidal capacity of the mammalian host cell, as already reported for other antiparasitic drugs.^18^ As NPD‐0228 (**7 s**) is only active against intracellular forms of the tested strains, it can be hypothesized that **7 s** interferes with intracellular processes essential for *T*. *cruzi* multiplication and viability of the highly metabolic proliferative form.

## Conclusions

Following an in vitro phenotypic screen and the identification of a 4‐bromophenyldihydropyrazole dimer (**3**, Figure 1) as hit with micromolar anti‐*T*. *cruzi* activity, a series of analogues was synthesized with variations on the phenyl ring (**7 a**–**w**, Table 1). The resulting SAR study led to the identification of the 3‐bromo‐4‐methoxyphenyl dimer (**7 s**) as optimized hit with a pIC_50_ value of 6.4 and no apparent toxicity toward the human MRC‐5 cell line. Attempts to introduce heterocycles (**11 a**–**f**, Table 2) instead of the phenyl moiety resulted in mostly inactive compounds, whereas changing the linker length (**12 a**–**e**, **14**, and **15**; Table 3) generally led to a decrease in activity. Testing of NPD‐0228 (**7 s**) on different strains revealed that the bromophenyldihydropyrazole dimer is inactive against the bloodstream (trypomastigote) form of the Y‐strain, while having sub‐micromolar potency on the intracellular (amastigote) forms of the different parasite strains that are clinically relevant for human infection. As benznidazole (**1**) is most active against the bloodstream form, NPD‐0228 (**7 s**) might be interesting to try in combination treatments. However, for this series to continue further, druglike properties must be improved substantially, as the current cLog*P* (6.1) and molecular weight (606 Da) are far from optimal.

## Experimental Section

### Biology

***T. cruzi*** **cellular assay**: Bloodstream trypomastigotes (BT) of the Y‐strain of *T*. *cruzi* were obtained by cardiac puncture of infected Swiss Webster mice on the parasitemia peak.^19^, ^20^ For the standard in vitro susceptibility assay on intracellular amastigotes, *T*. *cruzi* Tulahuen CL2, β‐galactosidase strain (nifurtimox‐sensitive) was used. The strain was maintained on MRC‐5_SV2_ (human lung fibroblast) cells in MEM, supplemented with 200 mm l‐glutamine, 16.5 mm NaHCO_3_, and 5 % inactivated fetal calf serum (FCS). All cultures and assays were conducted at 37 °C under an atmosphere of 5 % CO_2_.^14^

**MRC‐5 cytotoxicity cellular assay**: MRC‐5_SV2_ cells were cultivated in MEM, supplemented with l‐glutamine (20 mm), 16.5 mm sodium hydrogen carbonate and 5 % FCS. For the assay, 10^4^ cells per well were seeded onto 96‐well test plates containing the pre‐diluted sample and incubated at 37 °C and 5 % CO_2_ for 72 h. Cell viability was assessed fluorometrically 4 h after addition of resazurin (excitation 550 nm, emission 590 nm). The results are expressed as percentage decrease in cell viability relative to untreated controls.^14^

**Cytotoxicity assays on cardiac cells**: Non‐infected cultures were incubated at 37 °C up to 48 h with increasing concentrations of each compound diluted in RPMI. Morphology and spontaneous contractibility were evaluated by light microscopy and cellular viability was determined by PrestoBlue® tests.^21^ The results are expressed as the difference in decrease between treated and non‐treated cells according to the manufacturer′s instructions, and the LC_50_ value (minimum concentration that decreases cell viability by 50 %) was determined.^21^

**Trypanocidal activity**: Intracellular amastigotes of the *T*. *cruzi* strain were screened according to a methodology previously reported by Romanha et al.^22^ Bloodstream forms from the Y‐strain (5×10^6^ mL^−1^) obtained from Swiss male infected donor mice (5×10^4^ per animal, i.p.) at the parasitemia peak were incubated for 24 h at 37 °C in RPMI in the presence or absence of 1:3 serial dilutions of the compounds (0–50 μm) to determine parasite death rates through the direct quantification of live parasites by light microscopy. The IC_50_ (compound concentration that decreases the number of parasites by 50 %) was calculated. Selected compounds (IC_50_≤benznidazole (**1**)) were evaluated on intracellular amastigotes of the Y‐strain in infected cardiac cell cultures.^19^ After 24 h (10:1 parasite/cell infection ratio), the cultures were treated for 48 h at 37 °C with nontoxic concentrations of the compounds. Following treatment, the cultures were washed with saline, fixed with Bouin solution for 5 min, stained with Giemsa stain and evaluated by light microscopy. The percentage of infected host cells and the number of parasites per cell were determined for the calculation of the infection index and the pIC_50_ values.^20^ Selectivity index (SI) is expressed by the ratio between pLC_50_ (toxicity for mammalian cells) and the pIC_50_ (activity on the parasite). At least two independent assays were done in duplicate. In some assays, L929 cell cultures were incubated for 24 h at the corresponding IC_50_ value of NPD‐0228 (**7 s**), the cultures were rinsed using RPMI to remove the compound, and then infected and processed as described above.^22^ All procedures followed the guidelines as established by the Fiocruz Committee of Ethics for the Use of Animals (CEUA LW16/14).

### Chemistry

Chemicals and reagents were obtained from commercial suppliers and were used without further purification. Anhydrous DMF, THF, and CH_2_Cl_2_ were obtained by passing them through an activated alumina column prior to use. Microwave reactions were executed using a Biotage® Initiator microwave system. ^1^H NMR spectra were recorded on a Bruker Avance 250 (250 MHz), Bruker Avance 400 (400 MHz), Bruker Avance 500 (500 MHz) or Bruker 600 Avance (600 MHz) spectrometer. Data are reported as follows: chemical shift, integration, multiplicity (s=singlet, d=doublet, dd=double doublet, t=triplet, dt=double triplet, q=quartet, p=pentet, h=heptet, bs=broad singlet, m=multiplet), and coupling constants (Hz). Chemical shifts are reported in ppm with the natural abundance of deuterium in the solvent as the internal reference (CDCl_3_: *δ*=7.26 ppm, (CD_3_)_2_SO: *δ*=2.50 ppm). ^13^C NMR spectra were recorded on a Bruker Avance 500 (126 MHz) or Bruker Avance 600 (150 MHz). Chemical shifts are reported in ppm with the solvent resonance resulting from incomplete deuteration as the internal reference (CDCl_3_: *δ*=77.16 ppm or (CD_3_)_2_SO: *δ*=39.52 ppm). Systematic names for molecules according to IUPAC rules were generated using the ChemDraw AutoName program. LC–MS data were gathered using a Shimadzu HPLC–MS workstation with a LC‐20AD pump system, SPD‐M20A diode array detection, and a LC–MS 2010 EV mass spectrometer. The column used was an XBridge C18 5 μm column (100 mm×4.6 mm). Solvents used were the following: solvent B=MeCN, 0.1 % formic acid; solvent A=water, 0.1 % formic acid. The analysis was conducted using a flow rate of 1.0 mL min^−1^, start 5 % B, linear gradient to 90 % B in 4.5 min, then 1.5 min at 90 % B, linear gradient to 5 % B in 0.5 min, and then 1.5 min at 5 % B; total run time of 8 min. All reported compounds have purities >95 %, measured at 254 nm, unless otherwise mentioned. All HRMS spectra were recorded on a Bruker microTOF mass spectrometer using ESI in positive‐ion mode. Column purifications were either carried out automatically using Biotage equipment or manually, using 60–200 mesh silica. TLC analyses were performed with Merck F_254_ alumina silica plates using UV visualization. All reactions were done under N_2_ atmosphere, unless specifically mentioned.

#### General methods

**General method I: synthesis of β‐keto esters**: Benzoic acid **4 s** (25.0 g, 108 mmol) was suspended in CH_2_Cl_2_ (50 mL) while cooling to 0 °C. Subsequently, oxalyl dichloride (13.7 mL, 162 mmol) and DMF (0.08 mL, 1.08 mmol) were added, and the mixture was allowed to warm to room temperature. The mixture was stirred for 2 h, after which volatiles were evaporated. The remaining solids were dissolved in 50 mL THF. In a separate flask, methyl isobutyrate (18.6 mL, 162 mmol) was stirred in THF (50 mL) at −78 °C and a 2 m LDA (65 mL, 130 mmol) was added dropwise while maintaining −78 °C. Upon full addition, the mixture was stirred for 45 min, after which the acid chloride of **6** in THF was added dropwise, again maintaining the temperature at −78 °C. The reaction was allowed to warm to room temperature, after which the crude was quenched with water and extracted with diethyl ether. The organic phase was washed twice with water and once with brine. The organic layer was then dried with MgSO_4_, filtered, and evaporated to dryness. The crude was used in the next step without further purification.

**General method II: ring closure of β‐keto esters**: Crude keto ester **5 s** (34 g, 79 mmol) was dissolved in ethanol (75 mL) and hydrazine hydrate (64 %; 38.6 mL, 793 mmol) was added. The reaction was stirred at room temperature for 48 h, after which a white precipitate was visible. To the stirred solution 20 mL of water was added to allow further precipitation, after which solids were filtered off. Collected solids were dried in vacuo, yielding the desired product.

**General method III: installing the methylene linker**: Pyrazolone **6 s** (2.0 g, 6.73 mmol) was added to a microwave vial followed by TBAB (0.11 g, 0.34 mmol), 12 m NaOH (3 mL), and CH_2_Cl_2_ (8 mL), resulting in a white/yellow suspension. The vial was sealed and held at reflux in a sand bath at 60 °C overnight, after which the reaction mixture became a clear solution. The solution was diluted with CH_2_Cl_2_ (30 mL), and the organic layer was extracted with water (3×30 mL). The organic layer was dried over MgSO_4_, and the solvent was evaporated under vacuum to give a white/yellow solid. The obtained solids were recrystallized from MeOH.

**General method IV: installing aliphatic linkers**: Pyrazolone **6 s** (1.0 g, 3.4 mmol) was added to a flask and DMF (14 mL) was added, after which the mixture was cooled to 0 °C. Subsequently, NaH (60 % in mineral oil; 0.14 g, 3.4 mmol) was added. The reaction mixture was stirred at room temperature for 30 min, after which 1,2‐dibromoethane (0.145 mL, 1.68 mmol) was added dropwise at 0 °C. The reaction was stirred overnight at room temperature. Upon completion, the reaction was quenched with saturated aqueous NH_4_Cl and extracted with EtOAc (50 mL). The organic layer was washed with water (50 mL) and brine (40 mL). After this, the organic layer was dried over MgSO_4_ and volatiles were evaporated under vacuum. The crude product was purified over SiO_2_ using a gradient from 20 % EtOAc in *n*‐heptane toward 65 % EtOAc to give the title compound as an off‐white solid.

#### Experimental data

**5‐(3‐Bromo‐4‐methoxyphenyl)‐4,4‐dimethyl‐2,4‐dihydro‐3*H*‐pyrazol‐3‐one (6 s)**: Prepared according to general methods I and II using 25 g (43.3 mmol) of 3‐bromo‐4‐methoxybenzoic acid. The formed precipitate was filtered off and dried at 40 °C in vacuo to give 25.4 g (85.0 mmol, 79 %) of the title compound as a white solid. ^1^H NMR (500 MHz, [D_6_]DMSO): *δ*=11.54 (s, 1 H), 7.96 (d, *J=*2.2 Hz, 1 H), 7.77 (dd, *J=*8.6, 2.2 Hz, 1 H), 7.16 (d, *J=*8.7 Hz, 1 H), 3.89 (s, 3 H), 1.34 ppm (s, 6 H); ^13^C NMR (126 MHz, DMSO): *δ*=181.0, 160.6, 156.8, 130.3, 127.3, 125.3, 113.1, 111.8, 56.9, 46.8, 22.3 ppm; LC–MS (ESI) *m*/*z* found: 297 [*M*+H]^+^; *t*_R_: 4.51 min.

**2,2′‐Methylenebis(5‐(3‐bromo‐4‐methoxyphenyl)‐4,4‐dimethyl‐2,4‐dihydro‐3*H*‐pyrazol‐3‐one) (7 s; NPD‐0228)**: Prepared according to general method III using 2.0 g (6.7 mmol) of pyrazolone **6 s**. The white solid obtained was recrystallized from MeOH to give 1.8 g (3.0 mmol, 88 %) of the title compound as a white solid. ^1^H NMR (600 MHz, CDCl_3_): *δ*=7.98 (d, *J=*2.1 Hz, 2 H), 7.68 (dd, *J=*2.1, 8.7 Hz, 2 H), 6.89 (d, *J=*8.7 Hz, 2 H), 5.67 (s, 2 H), 3.92 (s, 6 H), 1.50 ppm (s, 12 H); ^13^C NMR (151 MHz, CDCl_3_): *δ*=179.2, 161.3, 157.4, 131.5, 126.9, 124.7, 112.4, 111.7, 56.5, 51.9, 48.4, 22.8 ppm; LC–MS (ESI) *m*/*z* found: 605 [*M*+H]^+^; *t*_R_: 5.23 min; HRMS‐ESI [*M*+H]^+^ calculated for C_25_H_27_Br_2_N_4_O_4_^+^: 605.0321, found: 605.0394.

**1,1′‐(Propane‐1,3‐diyl)bis(3‐(3‐bromo‐4‐methoxyphenyl)‐4,4‐dimethyl‐1*H*‐pyrazol‐5(4*H*)‐one) (12 b)**: Prepared according to general method IV using 1.0 g (3.37 mmol) of pyrazolone **6 a** and 0.171 mL (1.68 mmol) of 1,2‐dibromopropane. The crude was purified over SiO_2_ using a gradient from 20 % EtOAc in *n*‐heptane toward 60 % EtOAc to give 0.90 g (0.66 mmol, 84 %) of the title compound as a white solid. ^1^H NMR (600 MHz, CDCl_3_): *δ*=8.03 (d, *J=*2.1 Hz, 2 H), 7.69 (dd, *J=*2.1, 8.6 Hz, 2 H), 6.91 (d, *J=*8.7 Hz, 2 H), 3.94 (s, 6 H), 3.83 (t, *J=*7.0 Hz, 4 H), 2.26 (p, 7.0 Hz, 2 H), 1.48 ppm (s, 12 H); ^13^C NMR (151 MHz, CDCl_3_): *δ*=178.6, 160.9, 157.4, 131.6, 126.9, 125.3, 112.7, 111.9, 56.7, 48.7, 41.8, 27.3, 23.0 ppm; LC–MS (ESI) *m*/*z* found: 633 [*M*+H]^+^; *t*_R_: 5.45 min; HRMS‐ESI [*M*+H]^+^ calculated for C_27_H_31_Br_2_N_4_O_4_^+^: 633.0634, found: 633.0707.

**2,2′‐(Oxybis(ethane‐2,1‐diyl))bis(5‐(3‐bromo‐4‐methoxyphenyl)‐4,4‐dimethyl‐2,4‐dihydro‐3*H*‐pyrazol‐3‐one) (12 e)**: To mixture of pyrazolone **6 a** (200 mg, 0.67 mmol) and KOH (28.3 mg, 0.51 mmol) in dioxane (3 mL) was added 1‐bromo‐2‐(2‐bromoethoxy)ethane (78 mg, 0.334 mmol). The reaction mixture was heated at 110 °C for 18 h in the microwave. Upon completion the reaction was quenched with saturated aqueous NH_4_Cl and extracted with EtOAc. The organic layer was washed with water and brine. After this, the organic later was dried over MgSO_4_ and volatiles were evaporated under vacuum. The crude product was purified over SiO_2_ using a gradient from 30 % EtOAc in *n*‐heptane toward 80 % EtOAc to give 99 mg (0.15 mmol, 45 %) of the title compound as a white solid. ^1^H NMR (600 MHz, CDCl_3_): *δ*=8.00 (d, *J=*2.1 Hz, 2 H), 7.65 (dd, *J=*2.1, 8.6 Hz, 2 H), 6.89 (d, *J=*8.7 Hz, 2 H), 3.93 (s, 6 H), 3.91 (t, *J=*5.9 Hz, 4 H), 3.80 (t, *J=*5.9 Hz, 4 H), 1.44 ppm (s, 12 H); ^13^C NMR (151 MHz, CDCl_3_): *δ*=178.7, 160.4, 157.0, 131.2, 126.6, 124.9, 112.3, 111.6, 67.6, 56.4, 48.3, 43.8, 22.6 ppm; LC–MS (ESI) *m*/*z* found: 663 [*M*+H]^+^; *t*_R_: 5.24 min; HRMS‐ESI [*M*+H]^+^ calculated for C_28_H_33_Br_2_N_4_O_5_^+^: 663.0739, found: 663.0813.

**5‐(3‐Bromo‐4‐methoxyphenyl)‐4,4‐dimethyl‐2‐(oxiran‐2‐ylmethyl)‐2,4‐dihydro‐3*H*‐pyrazol‐3‐one (13)**: Pyrazolone **6 s** (2.0 g, 6.7 mmol), TBAB (0.18 g, 0.34 mmol), 50 % aqueous NaOH (4 mL) and 2‐(chloromethyl)oxirane (8 mL, 102 mmol) were added to a microwave tube resulting in a white suspension. The mixture was held at reflux (60 °C) overnight, after which the reaction mixture was a clear suspension. The two layers were separated, and the organic layer was extracted with water (3×10 mL). The organic layer was dried over MgSO_4_ and the solvent evaporated under vacuum to give a colorless oil. The obtained oil was purified over SiO_2_ using a gradient from 20 % EtOAc in *n*‐heptane toward 60 % EtOAc to give 1.22 g (3.47 mmol, 52 %) of the title compound as a colorless oil. ^1^H NMR (300 MHz, CDCl_3_): *δ*=8.04 (d, *J=*2.1 Hz, 1 H), 7.69 (dd, *J=*2.1, 8.6 Hz, 1 H), 6.92 (d, *J=*8.7 Hz, 1 H), 3.96–3.95 (m, 2 H), 3.94 (s, 3 H), 3.32–3.24 (m, 1 H), 2.86 (t, *J=*4.4 Hz, 1 H), 2.70 (dd, *J=*2.5, 4.8 Hz, 1 H), 1.49 ppm (s, 6 H); LC–MS (ESI) *m*/*z* found: 353 [*M*+H]^+^; *t*_R_: 4.23 min.

**1,1′‐(2‐Hydroxypropane‐1,3‐diyl)bis(3‐(3‐bromo‐4‐methoxyphenyl)‐4,4‐dimethyl‐1*H*‐pyrazol‐5(4*H*)‐one) (14)**: Pyrazolone **6 a** (1.0 g, 3.47 mmol), oxirane **20** (1.22 g, 3.47 mmol), and K_2_CO_3_ (1.44 g, 10.4 mmol) in DMF (0.2 m) were added to a microwave tube. The mixture was stirred at 100 °C overnight. Upon completion, the reaction mixture was allowed to cool to RT. The reaction was diluted in EtOAc and washed with water and brine. After this the organic layer was dried over MgSO_4_ and volatiles were evaporated under vacuum. The obtained solids were recrystallized from MeOH to give 1.2 g (1.9 mmol, 53 %) of the title compound as a white solid. ^1^H NMR (600 MHz, CDCl_3_): *δ*=8.00 (d, *J=*1.9 Hz, 2 H), 7.67 (dd, *J=*1.9, 8.6 Hz, 2 H), 6.91 (d, *J=*8.7 Hz, 2 H), 4.44 (p *J=*6.4 Hz, 1 H), 4.03–3.83 (m, 10 H), 1.51 (s, 6 H), 1.49 ppm (s, 6 H); ^13^C NMR (151 MHz, CDCl_3_): *δ*=179.2, 161.3, 157.4, 131.4, 126.8, 124.6, 112.5, 111.7, 69.1, 56.5, 48.5, 48.1, 22.8 ppm; LC–MS (ESI) *m*/*z* found: 649 [*M*+H]^+^; *t*_R_: 4.97 min; HRMS‐ESI [*M*+H]^+^ calculated for C_27_H_31_Br_2_N_4_O_5_^+^: 649.0583, found: 649.0656.

**1,1′‐(2‐Oxopropane‐1,3‐diyl)bis(3‐(3‐bromo‐4‐methoxyphenyl)‐4,4‐dimethyl‐1*H*‐pyrazol‐5(4*H*)‐one) (15)**: To a solution of alcohol **18** (200 mg, 0.31 mmol) in CH_2_Cl_2_ (1.5 mL), Dess–Martin periodinane (157 mg, 0.37 mmol) was added. The mixture was stirred for 2 h at RT. Next, diethyl ether (5 mL), saturated aqueous Na_2_S_2_O_3_ (5 mL), and saturated aqueous NaHCO_3_ (5 mL) were added, and the resulting mixture was stirred for 5 min. The organic phase was separated, dried over MgSO_4_, and volatiles were evaporated under vacuum. The reaction crude was purified over SiO_2_ using a gradient from 40 % EtOAc in *n*‐heptane toward 80 % EtOAc and recrystallized from MeOH to give 80 mg (0.12 mmol, 40 %) of the title compound as a white solid. ^1^H NMR (600 MHz, CDCl_3_): *δ*=8.01 (d, *J=*2.1 Hz, 2 H), 7.69 (dd, *J=*2.1, 8.6 Hz, 2 H), 6.92 (d, *J=*8.7 Hz, 2 H), 4.67 (s, 4 H), 3.94 (s, 6 H), 1.52 ppm (s, 12 H); ^13^C NMR (151 MHz, CDCl_3_): *δ*=197.6, 179.1, 161.5, 157.4, 131.6, 126.9, 124.6, 112.4, 111.8, 56.5, 51.7, 48.2, 22.8 ppm; LC–MS (ESI) *m*/*z* found: 647 [*M*+H]^+^; *t*_R_: 5.13 min; HRMS‐ESI [*M*+H]^+^ calculated for C_27_H_29_Br_2_N_4_O_5_^+^: 647.0426, found: 647.0499.

##### Contributions

*M.S., A.R.L., L.S., and K.O.: chemistry experiments; M.S., A.R.L., M.d.N.C.S.: writing of the initial manuscript, delivering figures and schemes; J.S. de A., A.M.: in vitro screening and phenotypic experiments; L.M., M.d.N.C.S., G.J.S, I.J.P.d.E., R.L.: supervision of the project, design of experiments, funding. All authors contributed to, and have read the final manuscript*.

## Conflict of interest

*The authors declare no conflict of interest*.

## Supporting information

As a service to our authors and readers, this journal provides supporting information supplied by the authors. Such materials are peer reviewed and may be re‐organized for online delivery, but are not copy‐edited or typeset. Technical support issues arising from supporting information (other than missing files) should be addressed to the authors.

Supplementary
