# Supplementary material for: Identification of Phenylpyrazolone Dimers as a New Class of Anti‐Trypanosoma cruzi Agents
Source: ChemMedChem. 2019 Aug 23;14(18):1662–8. doi: 10.1002/cmdc.201900370 (PMC6771560; doi:10.1002/cmdc.201900370)
Supplement: Supplementary file 1 — Supplementary [file CMDC-14-1662-s001.pdf]

CHEMMEDCHEM
Chemistry & Drug Discovery

Supporting Information

Identification of Phenylpyrazolone Dimers as a New Class of Anti-*Trypanosoma cruzi* Agents

Maarten Sijm,[a] Julianna Siciliano de Araújo,[b] Alba Ramos Llorca,[a] Kristina Orrling,[a] Lydia Stiny,[a] An Matheeussen,[c] Louis Maes,[c] Iwan J. P. de Esch,[a] Maria de Nazaré Correia Soeiro,[b] Geert Jan Sterk,[a] and Rob Leurs*[a]

cmdc_201900370_sm_miscellaneous_information.pdf

Author Contributions

M.S., A.R.L., L.S., and K.O.: chemistry experiments; M.S., A.R.L., M.d.N.C.S.: writing of the initial manuscript, delivering figures and schemes; J.S. de A., A.M.: in vitro screening and phenotypic experiments; L.M., M.d.N.C.S., G.J.S, I.J.P.d.E., R.L.: supervision of the project, design of experiments, funding. All authors contributed to, and have read the final manuscript.

Table of contents

Supporting information Table 1 5
General methods parasitology 6
General methods chemistry 6
General method I: synthesis of β-keto-esters 6
General method II: ring closure of β-keto-estes 6
General method III: Installing the methylene linker 7
General method IV: Installing aliphatic linkers 7
Experimental data 8
3-(3-bromophenyl)-4,4-dimethyl-1H-pyrazol-5(4H)-one (6a) 8
5-(2-bromophenyl)-4,4-dimethyl-2,4-dihydro-3H-pyrazol-3-one (6b) 8
4,4-dimethyl-5-phenyl-2,4-dihydro-3H-pyrazol-3-one (6c) 8
3-(4-chlorophenyl)-4,4-dimethyl-1H-pyrazol-5(4H)-one (6d) 9
5-(3-chlorophenyl)-4,4-dimethyl-2,4-dihydro-3H-pyrazol-3-one (6e) 9
5-(2-chlorophenyl)-4,4-dimethyl-2,4-dihydro-3H-pyrazol-3-one (6f) 9
3-(4-fluorophenyl)-4,4-dimethyl-1H-pyrazol-5(4H)-one (6g) 9
3-(3-fluorophenyl)-4,4-dimethyl-1H-pyrazol-5(4H)-one (6h) 10
5-(2-fluorophenyl)-4,4-dimethyl-2,4-dihydro-3H-pyrazol-3-one (6i) 10
3-(4-methoxyphenyl)-4,4-dimethyl-1H-pyrazol-5(4H)-one (6j) 10
3-(3-methoxyphenyl)-4,4-dimethyl-1H-pyrazol-5(4H)-one (6k) 10
5-(2-methoxyphenyl)-4,4-dimethyl-2,4-dihydro-3H-pyrazol-3-one (6l) 11
5-(4-(dimethylamino)phenyl)-4,4-dimethyl-2,4-dihydro-3H-pyrazol-3-one (6m) 11
5-(3-(dimethylamino)phenyl)-4,4-dimethyl-2,4-dihydro-3H-pyrazol-3-one (6n) 11
4-(4,4-dimethyl-5-oxo-4,5-dihydro-1H-pyrazol-3-yl)benzonitrile (6o) 12
3-(4,4-dimethyl-5-oxo-4,5-dihydro-1H-pyrazol-3-yl)benzonitrile (6p) 12
5-(3-fluoro-4-methoxyphenyl)-4,4-dimethyl-2,4-dihydro-3H-pyrazol-3-one (6q) 12
5-(4-chloro-3-methoxyphenyl)-4,4-dimethyl-2,4-dihydro-3H-pyrazol-3-one (6r) 12
5-(3-chloro-4-methoxyphenyl)-4,4-dimethyl-2,4-dihydro-3H-pyrazol-3-one (6t) 13
5-(4-bromo-3-methoxyphenyl)-4,4-dimethyl-2,4-dihydro-3H-pyrazol-3-one (6u) 13
3-(3,4-dimethoxyphenyl)-4,4-dimethyl-1H-pyrazol-5(4H)-one (6v) 13
3-(3,4-difluorophenyl)-4,4-dimethyl-1H-pyrazol-5(4H)-one (6w) 14
1,1'-methylenebis(3-(3-bromophenyl)-4,4-dimethyl-1H-pyrazol-5(4H)-one) (7a) 14
2,2'-methylenebis(5-(2-bromophenyl)-4,4-dimethyl-2,4-dihydro-3H-pyrazol-3-one) (7b) 14
2,2'-methylenebis(4,4-dimethyl-5-phenyl-2,4-dihydro-3H-pyrazol-3-one) (7c) 15
1,1'-methylenebis(3-(4-chlorophenyl)-4,4-dimethyl-1H-pyrazol-5(4H)-one) (7d) 15
2,2'-methylenebis(5-(3-chlorophenyl)-4,4-dimethyl-2,4-dihydro-3H-pyrazol-3-one) (7e). 15

2,2'-methylenebis(5-(2-chlorophenyl)-4,4-dimethyl-2,4-dihydro-3H-pyrazol-3-one) (7f) . 16
1,1'-methylenebis(3-(4-fluorophenyl)-4,4-dimethyl-1H-pyrazol-5(4H)-one) (7g) 16
1,1'-methylenebis(3-(3-fluorophenyl)-4,4-dimethyl-1H-pyrazol-5(4H)-one) (7h) 16
2,2'-methylenebis(5-(2-fluorophenyl)-4,4-dimethyl-2,4-dihydro-3H-pyrazol-3-one) (7i).. 17
1,1'-methylenebis(3-(4-methoxyphenyl)-4,4-dimethyl-1H-pyrazol-5(4H)-one) (7j) 17
1,1'-methylenebis(3-(3-methoxyphenyl)-4,4-dimethyl-1H-pyrazol-5(4H)-one) (7k) 17
2,2'-methylenebis(5-(2-methoxyphenyl)-4,4-dimethyl-2,4-dihydro-3H-pyrazol-3-one) (7l) 18
2,2'-methylenebis(5-(4-(dimethylamino)phenyl)-4,4-dimethyl-2,4-dihydro-3H-pyrazol-3-one) (7m) 18
2,2'-methylenebis(5-(3-(dimethylamino)phenyl)-4,4-dimethyl-2,4-dihydro-3H-pyrazol-3-one) (7n) 18
4,4'-(methylenebis(4,4-dimethyl-5-oxo-4,5-dihydro-1H-pyrazole-1,3-diyl))dibenzonitrile (7o) 19
3,3'-(methylenebis(4,4-dimethyl-5-oxo-4,5-dihydro-1H-pyrazole-1,3-diyl))dibenzonitrile (7p) 19
2,2'-methylenebis(5-(3-fluoro-4-methoxyphenyl)-4,4-dimethyl-2,4-dihydro-3H-pyrazol-3-one) (7q) 19
2,2'-methylenebis(5-(3-chloro-4-methoxyphenyl)-4,4-dimethyl-2,4-dihydro-3H-pyrazol-3-one) (7r) 20
2,2'-methylenebis(5-(4-chloro-3-methoxyphenyl)-4,4-dimethyl-2,4-dihydro-3H-pyrazol-3-one) (7t) 20
2,2'-methylenebis(5-(4-bromo-3-methoxyphenyl)-4,4-dimethyl-2,4-dihydro-3H-pyrazol-3-one) (7u) 20
1,1'-methylenebis(3-(3,4-dimethoxyphenyl)-4,4-dimethyl-1H-pyrazol-5(4H)-one) (7v) ... 21
1,1'-methylenebis(3-(3,4-difluorophenyl)-4,4-dimethyl-1H-pyrazol-5(4H)-one) (7w) 21
4,4-dimethyl-3-(thiophen-2-yl)-1H-pyrazol-5(4H)-one (10a) 21
4,4-dimethyl-5-(thiophen-3-yl)-2,4-dihydro-3H-pyrazol-3-one (10b) 21
5-(furan-2-yl)-4,4-dimethyl-2,4-dihydro-3H-pyrazol-3-one (10c) 22
3-(furan-3-yl)-4,4-dimethyl-1H-pyrazol-5(4H)-one (10d) 22
4,4-dimethyl-3-(thiazol-4-yl)-1H-pyrazol-5(4H)-one (10e) 22
5-(5-bromothiophen-2-yl)-4,4-dimethyl-2,4-dihydro-3H-pyrazol-3-one (10f) 22
4,4-dimethyl-2,4-dihydro-3H-pyrazol-3-one (10g) 23
1,1'-methylenebis(4,4-dimethyl-3-(thiophen-2-yl)-1H-pyrazol-5(4H)-one) (11a) 23
2,2'-methylenebis(4,4-dimethyl-5-(thiophen-3-yl)-2,4-dihydro-3H-pyrazol-3-one) (11b) . 23
2,2'-methylenebis(5-(furan-2-yl)-4,4-dimethyl-2,4-dihydro-3H-pyrazol-3-one) (11c) 24
2,2'-methylenebis(5-(furan-3-yl)-4,4-dimethyl-2,4-dihydro-3H-pyrazol-3-one) (11d) 24
2,2'-methylenebis(4,4-dimethyl-5-(thiazol-4-yl)-2,4-dihydro-3H-pyrazol-3-one) (11e) 24
2,2'-methylenebis(5-(5-bromothiophen-2-yl)-4,4-dimethyl-2,4-dihydro-3H-pyrazol-3-one) (11f) 25

2,2'-methylenebis(4,4-dimethyl-2,4-dihydro-3H-pyrazol-3-one) (11g) 25
1,1'-(ethane-1,2-diyl)bis(3-(3-bromo-4-methoxyphenyl)-4,4-dimethyl-1H-pyrazol-5(4H)-one) (12a) 25
1,1'-(butane-1,4-diyl)bis(3-(3-bromo-4-methoxyphenyl)-4,4-dimethyl-1H-pyrazol-5(4H)-one) (12c) 26
References 27

Supporting information Table 1

SI Table 1 Phenotypic activity against *T. brucei*, *T. cruzi*, *L. infantum* and cytotoxicity against human MRC-5 cells

Cmpnd	pIC_{50} Tbr[a]	pIC_{50} Tcr[a]	pIC_{50} Linf[a]	pIC_{50} MRC-5[a]	Cmpnd	pIC_{50} Tbr[a]	pIC_{50} Tcr[a]	pIC_{50} Linf[a]	pIC_{50} MRC-5[a]
**3**	5.1	5.4	5.1	< 4.2	**7s**	4.9	6.4	4.8	< 4.2
**7a**	< 4.2	< 4.2	< 4.2	< 4.2	**7t**	5.1	< 4.2	4.9	< 4.2
**7b**	< 4.2	< 4.2	4.5	< 4.2	**7u**	< 4.2	< 4.2	5.2	< 4.2
**7c**	5.1	5.3	4.8	5.1	**7v**	< 4.2	< 4.3	< 4.2	< 4.2
**7d**	< 4.2	< 4.2	< 4.2	< 4.2	**7w**	4.5	5.1	< 4.2	< 4.2
**7e**	< 4.2	< 4.2	< 4.2	< 4.2	**11a**	< 4.2	< 4.2	< 4.2	< 4.2
**7f**	< 4.2	< 4.2	< 4.2	< 4.2	**11b**	< 4.2	< 4.2	< 4.2	< 4.2
**7g**	4.4	5.2	< 4.2	< 4.2	**11c**	< 4.2	< 4.2	< 4.2	< 4.2
**7h**	4.5	4.3	< 4.2	< 4.2	**11d**	< 4.2	< 4.2	< 4.2	< 4.2
**7i**	4.5	5.0	< 4.2	< 4.2	**11e**	< 4.2	< 4.2	< 4.2	< 4.2
**7j**	4.5	5.1	4.4	4.4	**11f**	5.1	5.2	5.2	< 4.2
**7k**	< 4.2	< 4.2	< 4.2	< 4.2	**11g**	< 4.2	< 4.2	< 4.2	< 4.2
**7l**	< 4.2	< 4.2	4.7	< 4.2	**12a**	< 4.2	< 4.2	< 4.2	< 4.2
**7m**	< 4.2	< 4.2	< 4.2	< 4.2	**12b**	< 4.2	< 4.2	< 4.2	< 4.2
**7n**	< 4.2	< 4.2	< 4.2	< 4.2	**12c**	< 4.2	< 4.2	< 4.2	< 4.2

a all reported values are within a standard deviation of ±0.2

General methods parasitology

The phenotypic assays against *T. brucei*, *T. cruzi*, *L. infantum* and cytotoxicity against MRC-5 cells were done as previously described in Blaazer et al.[1]

General methods chemistry

General method I: synthesis of β-keto-esters

Benzoic acid **4s** (25.0 g, 108 mmol) was suspended in DCM (50 mL) while cooling to 0°C. Subsequently oxalyl dichloride (13.7 mL, 162 mmol) and DMF (0.08 mL, 1.08 mmol) were added and the mixture was allowed to warm up to room temperature. The mixture was stirred for 2 h after which volatiles were evaporated. The remaining solids were redissolved in 50 ml of THF. In a separate flask methyl isobutyrate (18.6 mL, 162 mmol) was stirred in THF (50 mL) at -78°C and a 2M LDA (65 mL, 130 mmol) was added dropwise while maintaining -78°C. Upon full addition, the mixture was stirred for 45m after which the acid chloride of **6** in THF was added dropwise, again maintaining the temperate at -78°C. The reaction was allowed to warm up to room temperature after which crude was quenched with water and extracted with diethyl ether. The organic phase was washed twice with water and once with brine. The organic layer was then dried with $MgSO_4$, filtered and evaporated to dryness. The crude was used in the next step without further purification.

General method II: ring closure of β-keto-estes

Crude keto-ester **5s** (34 g, 79 mmol) was dissolved in ethanol (75 mL) and hydrazine hydrate (64%) (38.6 mL, 793 mmol) was added. The reaction was stirred at room temperature for 48h after which white precipitation was visible. To the stirred solution 20 mL of water was added

to allow further precipitation, after which solids were filtered off. Collected solids were dried *in vacuo* yielding the desired product.

General method III: Installing the methylene linker

Pyrazolone **6s** (2.0 g, 6.73 mmol), was added to a microwave vial followed by TBAB (0.11 g, 0.34 mmol), 12M NaOH (3 mL) and DCM (8 mL) resulting in a white/yellow suspension. The vial was sealed and refluxed in a sand bath at 60°C overnight after which the reaction mixture was a clear solution. The solution was diluted with DCM (30 mL), and the organic layer was extracted with water (3 x 30 mL). The organic layer was dried over $MgSO_4$ and the solvent evaporated under *vacuo* to give a white/yellow solid. The obtained solids were recrystallized from MeOH.

General method IV: Installing aliphatic linkers

Pyrazolone **6s** (1.0 g, 3.4 mmol) was added to a flask and DMF (14 mL) was added after which the mixture was cooled to 0°C. Subsequently, NaH (60% in mineral oil)(0.14 g, 3.4 mmol) was added. The reaction mixture was stirred at room temperature for 30 min, after which 1,2-dibromoethane (0.145 mL, 1.68 mmol) was added dropwise at 0°C. The reaction was stirred overnight at room temperature. Upon completion, the reaction was quenched with sat. aqueous NH_4Cl and extracted with EtOAc (50 mL). The organic layer was washed with water (50 mL) and brine (40 mL). After this, the organic layer was dried over $MgSO_4$ and volatiles were evaporated under *vacuo*. The crude product was purified over SiO_2 using a gradient from 20% EtOAc in n-heptane towards 65% EtOAc to give the title compound as an off-white solid.

Experimental data

The experimental data of **5s**, **6s**, **7s**, **12b**, **12e**, **13**, **14** and **15** can be found in the main text of this publication.

3-(3-bromophenyl)-4,4-dimethyl-1H-pyrazol-5(4H)-one (6a)

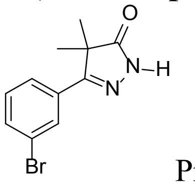

Prepared according to general method I and II using 2.0 g (9.9 mmol) of 3-bromobenzoic acid. Formed little precipitate, the crude was purified over SiO_2 using a gradient from 20% EtOAc in n-heptane towards 60% EtOAc to give 1.5 g (5.6 mmol, 57%) of the title compound as a yellow solid. ^{1}H NMR (300 MHz, DMSO-d_6) δ 11.68 (s, 1H), 7.93 (t, J = 1.6 Hz, 1H), 7.87 – 7.77 (m, 1H), 7.73 – 7.59 (m, 1H), 7.42 (t, J = 7.9 Hz, 1H), 1.36 (s, 6H). ^{13}C NMR (151 MHz, DMSO-d_6) δ 180.7, 160.2, 133.1, 132.4, 131.1, 127.9, 124.9, 122.2, 46.4, 21.7. LC-MS (ESI) *m/z* found: no mass observed [M+H]$^+$; retention time: 4.03 minutes. HRMS-ESI [M+H]$^+$ calculated for $C_{11}H_{12}BrN_2O^+$: 267.0055, found: 267.0128.

5-(2-bromophenyl)-4,4-dimethyl-2,4-dihydro-3H-pyrazol-3-one (6b)

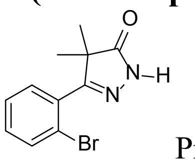

Prepared according to general method I and II using 2.0 g (9.9 mmol) of 2-bromobenzoic acid. Formed little precipitate, the crude was purified over SiO_2 using a gradient from 20% EtOAc in n-heptane towards 60% EtOAc to give 1.8 g (6.7 mmol, 68%) of the title compound as a white solid. ^{1}H NMR (300 MHz, DMSO) δ 11.57 (s, 1H), 7.80 – 7.73 (m, 1H), 7.54 – 7.38 (m, 3H), 1.18 (s, 6H). ^{13}C NMR (151 MHz, $CDCl_3$) δ 184.9, 167.8, 138.4, 137.8, 136.4, 136.0, 132.8, 127.8, 53.8, 25.9. LC-MS (ESI) *m/z* found: 267 [M+H]$^+$; retention time: 3.69 minutes. HRMS-ESI [M+H]$^+$ calculated for $C_{11}H_{12}BrN_2O^+$: 267.0055, found: 267.0128.

4,4-dimethyl-5-phenyl-2,4-dihydro-3H-pyrazol-3-one (6c)

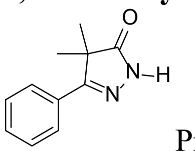

Prepared according to general method I and II using 2.0 g (16.4 mmol) of benzoic acid. Formed precipitate was filtered off dried at 40°C *in vacuo*. The obtained solids were recrystallized from MeOH to give 1.52 g (8.1 mmol, 49%) of the title compound as a white solid. ^{1}H NMR (300 MHz, DMSO) δ 11.54 (s, 1H), 7.84 – 7.78 (m, 2H), 7.50 – 7.40 (m, 3H), 1.36 (s, 6H). ^{13}C NMR (151 MHz, DMSO) δ 186.0, 166.8, 136.2, 134.9, 134.1, 131.0, 51.6, 27.1. LC-MS (ESI) *m/z* found: 189 [M+H]$^+$; retention time: 3.50 minutes. HRMS-ESI [M+H]$^+$ calculated for $C_{11}H_{12}N_2O^+$: 189.0950, found: 189.1022.

3-(4-chlorophenyl)-4,4-dimethyl-1H-pyrazol-5(4H)-one (6d)

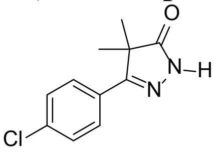

Prepared according to general method I and II using 2.0 g (12.8 mmol) of 4-chlorobenzoic acid. Formed precipitate was filtered off dried at 40°C *in vacuo.* The obtained solids were recrystallized from MeOH to give 1.73 g (7.8 mmol, 61%) of the title compound as a yellow solid. ^{1}H NMR (300 MHz, DMSO-d_6) δ 11.63 (s, 1H), 7.85 (d, *J* = 8.7 Hz, 2H), 7.52 (d, *J* = 8.7 Hz, 2H), 1.36 (s, 6H). ^{13}C NMR (151 MHz, DMSO-d_6) δ 180.7, 160.6, 134.2, 129.7, 128.9, 127.6, 46.3, 21.7. LC-MS (ESI) *m*/*z* found: 223 $[M+H]^+$; retention time: 3.97 minutes. HRMS-ESI $[M+H]^+$ calculated for $C_{11}H_{12}ClN_2O^+$: 223.0560, found: 223.0633.

5-(3-chlorophenyl)-4,4-dimethyl-2,4-dihydro-3H-pyrazol-3-one (6e)

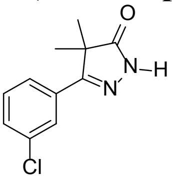

Prepared according to general method I and II using 2.0 g (12.8 mmol) of 3-chlorobenzoic acid. Formed little precipitate, the crude was purified over SiO_2 using a gradient from 20% EtOAc in n-heptane towards 60% EtOAc to give 0.93 g (4.2 mmol, 33%) of the title compound as a yellow solid. ^{1}H NMR (600 MHz, DMSO) δ 11.68 (s, 1H), 7.79 (s, 1H), 7.76 (dt, *J* = 1.5, 1.5, 7.0 Hz, 1H), 7.53 – 7.48 (m, 2H), 1.37 (s, 6H). ^{13}C NMR (151 MHz, DMSO) δ 180.3, 159.9, 133.4, 132.6, 130.5, 129.1, 124.7, 124.2, 46.0, 39.2, 21.4. LC-MS (ESI) *m*/*z* found: 223 $[M+H]^+$; retention time: 3.97 minutes. HRMS-ESI $[M+H]^+$ calculated for $C_{11}H_{12}ClN_2O^+$: 223.0560, found: 223.0633.

5-(2-chlorophenyl)-4,4-dimethyl-2,4-dihydro-3H-pyrazol-3-one (6f)

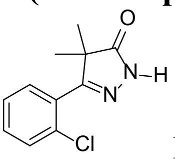

Prepared according to general method I and II using 2.0 g (12.8 mmol) of 2-chlorobenzoic acid. Formed precipitate was filtered off dried at 40°C *in vacuo.* The obtained solids were recrystallized from MeOH to give 2.1 g (9.4 mmol, 74%) of the title compound as a white solid. ^{1}H NMR (600 MHz, DMSO) δ 11.57 (s, 1H), 7.59 (d, *J* = 7.8 Hz, 1H), 7.50 (td, *J* = 2.7, 7.3, 8.1 Hz, 1H), 7.47 – 7.42 (m, 2H), 1.16 (s, 6H). ^{13}C NMR (151 MHz, DMSO) δ 180.2, 162.1, 133.1, 131.5, 131.4, 131.1, 130.4, 127.6, 49.0, 21.2. LC-MS (ESI) *m*/*z* found: 223 $[M+H]^+$; retention time: 3.62 minutes. HRMS-ESI $[M+H]^+$ calculated for $C_{11}H_{12}ClN_2O^+$: 223.0560, found: 223.0633.

3-(4-fluorophenyl)-4,4-dimethyl-1H-pyrazol-5(4H)-one (6g)

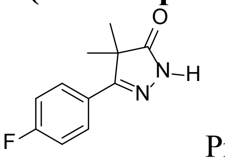

Prepared according to general method I and II using 2.0 g (14.3 mmol) of 4-fluorobenzoic acid. Formed little precipitate, the crude was purified over SiO_2 using a gradient from 20% EtOAc in n-heptane towards 60% EtOAc to give 1.79 g (8.68 mmol, 61%) of the title compound as a white solid. ^{1}H NMR (300 MHz, DMSO-d_6) δ 11.55 (s, 1H), 7.87 (dd, *J* = 8.9, 5.6 Hz, 2H), 7.28 (t, *J* = 8.9 Hz, 2H), 1.35 (s, 6H). ^{13}C NMR (151 MHz, DMSO) δ 180.8, 163.7, 162.1, 160.9, 128.3, 128.3, 127.7, 127.7, 116.1, 116.0, 46.5, 21.9. LC-MS (ESI) *m*/*z*

found: 207 [M+H]$^+$; retention time: 3.58 minutes. HRMS-ESI [M+H]$^+$ calculated for $C_{11}H_{12}FN_2O^+$: 207.0855, found: 207.0928.

3-(3-fluorophenyl)-4,4-dimethyl-1H-pyrazol-5(4H)-one (6h)

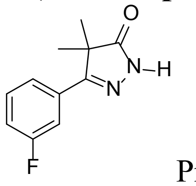

Prepared according to general method I and II using 2.0 g (14.3 mmol) of 3-fluorobenzoic acid. Formed little precipitate, the crude was purified over SiO_2 using a gradient from 20% EtOAc in n-heptane towards 60% EtOAc to give 1.32 g (6.4 mmol, 45%) of the title compound as a white solid. ^{1}H NMR (600 MHz, DMSO-d_6) δ 11.65 (s, 1H), 7.65 (d, J = 7.8 Hz, 1H), 7.57 (d, J = 10.5 Hz, 1H), 7.51 (q, J = 7.5 Hz, 1H), 7.29 (t, J = 8.5 Hz, 1H), 1.37 (s, 6H). ^{13}C NMR (151 MHz, DMSO) δ 180.9, 163.3, 161.7, 160.6, 160.6, 133.3, 133.3, 131.2, 131.2, 122.2, 122.2, 116.8, 116.6, 112.3, 112.2, 46.5, 21.9. LC-MS (ESI) *m/z* found: 207 [M+H]$^+$; retention time: 3.63 minutes. HRMS-ESI [M+H]$^+$ calculated for $C_{11}H_{12}FN_2O^+$: 207.0855, found: 207.0928.

5-(2-fluorophenyl)-4,4-dimethyl-2,4-dihydro-3H-pyrazol-3-one (6i)

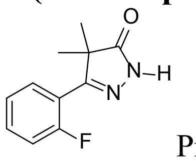

Prepared according to general method I and II using 2.0 g (14.3 mmol) of 2-fluorobenzoic acid. Formed precipitate was filtered off dried at 40°C *in vacuo.* The obtained solids were recrystallized from MeOH to give 1.42 g (6.9 mmol, 48%) of the title compound as a white solid. ^{1}H NMR (600 MHz, DMSO) δ 11.63 (s, 1H), 7.67 (td, J = 1.6, 7.7, 7.7 Hz, 1H), 7.52 (qd, J = 1.7, 7.2, 7.2, 7.2 Hz, 1H), 7.36 – 7.26 (m, 2H), 1.24 (s, 6H). ^{13}C NMR (151 MHz, DMSO) δ 180.7, 160.8, 159.7, 159.7, 159.1, 132.2, 132.1, 130.4, 130.4, 125.2, 125.2, 122.7, 122.6, 117.1, 116.9, 48.1, 21.4. LC-MS (ESI) *m/z* found: 207 [M+H]$^+$; retention time: 3.51 minutes. HRMS-ESI [M+H]$^+$ calculated for $C_{11}H_{12}FN_2O^+$: 207.0855, found: 207.0928.

3-(4-methoxyphenyl)-4,4-dimethyl-1H-pyrazol-5(4H)-one (6j)

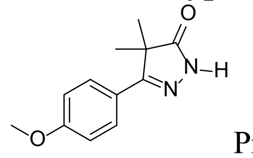

Prepared according to the general method I and II using 2.0 g (13.2 mmol) of 4-methoxybenzoic acid. The formed precipitate was filtered off dried at 40°C *in vacuo.* The obtained solids were recrystallized from MeOH to give 1.22 g (5.6 mmol, 46%) of the title compound as a white solid. ^{1}H NMR (300 MHz, DMSO) δ 11.39 (s, 1H), 7.76 (d, J = 8.7 Hz, 2H), 7.00 (d, J = 8.7 Hz, 2H), 3.80 (s, 3H), 1.34 (s, 6H). ^{13}C NMR (151 MHz, $CDCl_3$) δ 180.6, 161.4, 160.3, 127.4, 123.6, 114.3, 55.3, 46.4, 22.0. LC-MS (ESI) *m/z* found: 219 [M+H]$^+$; retention time: 5.23 minutes.

3-(3-methoxyphenyl)-4,4-dimethyl-1H-pyrazol-5(4H)-one (6k)

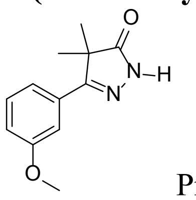

Prepared according to general method I and II using 0.5 g (3.5 mmol) of 3-methoxybenzoic acid. Formed little precipitate, the crude was purified over SiO_2 using a

gradient from 20% EtOAc in n-heptane towards 60% EtOAc to give 0.45 g (2.1 mmol, 60%) of the title compound as a white solid. ^{1}H NMR (600 MHz, DMSO-d_6) δ 11.55 (s, 1H), 7.38 – 7.37 (m, 1H), 7.37 – 7.36 (m, 1H), 7.31 – 7.29 (m, 1H), 7.04 – 7.01 (m, 1H), 3.79 (s, 3H), 1.36 (s, 6H). ^{13}C NMR (151 MHz, DMSO) δ 180.6, 161.3, 159.3, 132.1, 129.9, 118.2, 115.5, 110.4, 55.0, 46.3, 21.9. LC-MS (ESI) *m*/*z* found: 219 $[M+H]^+$; retention time: 3.52 minutes.

5-(2-methoxyphenyl)-4,4-dimethyl-2,4-dihydro-3H-pyrazol-3-one (6l)

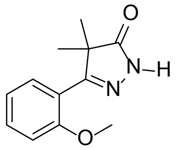

Prepared according to general method I and II using 2.0 g (13.2 mmol) of 2-methoxybenzoic acid. Formed little precipitate, the crude was purified over SiO_2 using a gradient from 20% EtOAc in n-heptane towards 60% EtOAc to give 1.9 g (8.7 mmol, 66%) of the title compound as a white solid. ^{1}H NMR (300 MHz, DMSO) δ 11.37 (s, 1H), 7.48 – 7.40 (m, 1H), 7.30 (d, *J* = 7.5 Hz, 1H), 7.12 (d, *J* = 8.3 Hz, 1H), 7.01 (t, *J* = 7.4, 7.4 Hz, 1H), 3.77 (s, 3H), 1.13 (s, 6H). ^{13}C NMR (151 MHz, $CDCl_3$) δ 185.7, 168.1, 162.6, 136.1, 135.5, 126.2, 125.5, 117.0, 60.5, 53.5, 26.3. LC-MS (ESI) *m*/*z* found: 219 $[M+H]^+$; retention time: 3.40 minutes.

5-(4-(dimethylamino)phenyl)-4,4-dimethyl-2,4-dihydro-3H-pyrazol-3-one (6m)

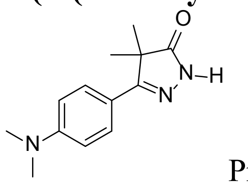

Prepared according to general method I and II using 2.0 g (12.1 mmol) of 4-(dimethylamino)benzoic acid Formed precipitate was filtered off dried at 40°C *in vacuo*. The obtained solids were recrystallized from MeOH to give 0.54 g (2.4 mmol, 2%) of the title compound as a white solid. ^{1}H NMR (300 MHz, DMSO-d_6) δ 11.22 (s, 1H), 7.64 (d, *J* = 8.9 Hz, 2H), 6.73 (d, *J* = 8.9 Hz, 2H), 2.95 (s, 6H), 1.33 (s, 6H). ^{13}C NMR (151 MHz, DMSO) δ 180.2, 161.7, 150.6, 126.5, 118.1, 111.4, 45.9, 39.4, 39.2, 21.9. LC-MS (ESI) *m*/*z* found: 232 $[M+H]^+$; retention time: 3.53 minutes. HRMS-ESI $[M+H]^+$ calculated for $C_{13}H_{18}N_3O^+$: 232.1372, found: 232.1444.

5-(3-(dimethylamino)phenyl)-4,4-dimethyl-2,4-dihydro-3H-pyrazol-3-one (6n)

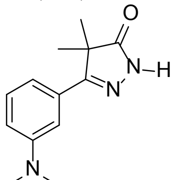

Prepared according to general method I and II using 2.0 g (12.1 mmol) of 3-(dimethylamino)benzoic acid. Formed little precipitate, the crude was purified over SiO_2 using a gradient from 20% EtOAc in n-heptane towards 60% EtOAc to give 0.90 g (3.9 mmol, 32%) of the title compound as a yellow solid. ^{1}H NMR (300 MHz, DMSO) δ 11.45 (s, 1H), 7.25 (t, *J* = 8.0, 8.0 Hz, 1H), 7.09 – 7.03 (m, 2H), 6.85 – 6.79 (m, 1H), 2.92 (s, 6H), 1.35 (s, 6H). ^{13}C NMR (151 MHz, $CDCl_3$) δ 180.8, 162.3, 150.5, 131.5, 129.3, 114.0, 108.9, 46.5, 40.1, 22.2. LC-MS (ESI) *m*/*z* found: 232 $[M+H]^+$; retention time: 3.11 minutes. HRMS-ESI $[M+H]^+$ calculated for $C_{13}H_{18}N_3O^+$: 232.1372, found: 232.1444.

4-(4,4-dimethyl-5-oxo-4,5-dihydro-1H-pyrazol-3-yl)benzonitrile (6o)

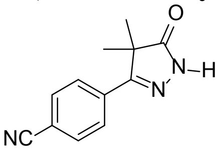

Prepared according to general method I and II using 2.0 g (13.6 mmol) of 4-cyanobenzoic acid. Formed little precipitate, the crude was purified over SiO_2 using a gradient from 20% EtOAc in n-heptane towards 60% EtOAc to give 0.68 g (3.2 mmol, 23%) of the title compound as a yellow solid. ^{1}H NMR (600 MHz, DMSO) δ 11.84 (s, 1H), 7.99 (d, J = 8.6 Hz, 2H), 7.90 (d, J = 8.6 Hz, 2H), 1.37 (s, 6H). ^{13}C NMR (151 MHz, DMSO) δ 180.6, 159.8, 134.7, 132.6, 126.2, 118.3, 111.5, 46.1, 39.2, 21.4. LC-MS (ESI) *m*/*z* found: no mass observed $[M+H]^+$; retention time: 3.39 minutes. HRMS-ESI $[M+H]^+$ calculated for $C_{12}H_{12}N_3O^+$: 214.0902, found: 214.0975.

3-(4,4-dimethyl-5-oxo-4,5-dihydro-1H-pyrazol-3-yl)benzonitrile (6p)

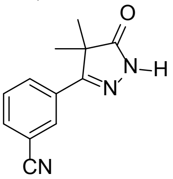

Prepared according to general method I and II using 2.0 g (13.6 mmol) of 3-cyanobenzoic acid. Formed little precipitate, the crude was purified over SiO_2 using a gradient from 20% EtOAc in n-heptane towards 60% EtOAc to give 0.55 g (2.6 mmol, 2%) of the title compound as a yellow solid. ^{1}H NMR (300 MHz, DMSO-d_6) δ 11.76 (s, 1H), 8.2 – 8.11 (m, 2H), 7.91 (dd, 1H), 7.67 (t, J = 7.8 Hz, 1H), 1.38 (s, 6H). ^{13}C NMR (151 MHz, $CDCl_3$) δ 180.7, 159.8, 133.0, 132.0, 130.2, 128.9, 118.3, 112.3, 46.3, 21.5. LC-MS (ESI) *m*/*z* found: no mass observed $[M+H]^+$; retention time: 3.35 minutes. HRMS-ESI $[M+H]^+$ calculated for $C_{12}H_{12}N_3O^+$: 214.0902, found: 214.0975.

5-(3-fluoro-4-methoxyphenyl)-4,4-dimethyl-2,4-dihydro-3H-pyrazol-3-one (6q)

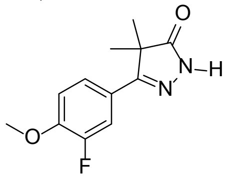

Prepared according to general method I and II using 3.0 g (17.6 mmol) of 4-fluoro-3-methoxybenzoic acid. The formed precipitate was filtered off dried at 40°C *in vacuo.* The obtained solids were recrystallized from MeOH to give 1.2 g (5.2 mmol, 29%) of the title compound as a white solid. ^{1}H NMR (600 MHz, DMSO) δ 11.50 (s, 1H), 7.62 – 7.56 (m, 2H), 7.22 (t, J = 8.9 Hz, 1H), 3.88 (s, 3H), 1.35 (s, 6H). ^{13}C NMR (151 MHz, DMSO) δ 180.6, 160.5, 152.3, 150.7, 148.3, 123.9, 122.8, 113.9, 112.8, 56.1, 46.4, 21.9. LC-MS (ESI) *m*/*z* found: 237 $[M+H]^+$; retention time: 3.55 minutes. HRMS-ESI $[M+H]^+$ calculated for $C_{12}H_{14}FN_2O_2^+$: 237.1034, found: 237.1042.

5-(4-chloro-3-methoxyphenyl)-4,4-dimethyl-2,4-dihydro-3H-pyrazol-3-one (6r)

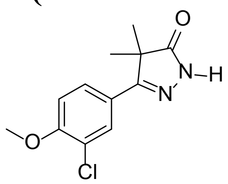

Prepared according to general method I and II using 2.0 g (10.7 mmol) of 3-chloro-4-methoxybenzoic acid. Formed little precipitate, the crude was purified over SiO_2 using a gradient from 20% EtOAc in n-heptane towards 60% EtOAc to give 0.45 g (1.8 mmol, 17%) of the title compound as a white solid. ^{1}H NMR (600 MHz, DMSO) δ 11.64 (s, 1H), 7.50

(d, J = 8.3 Hz, 1H), 7.46 (d, J = 1.9 Hz, 1H), 7.36 (dd, J = 1.9, 8.3 Hz, 1H), 3.92 (s, 3H), 1.37 (s, 6H). ^{13}C NMR (151 MHz, DMSO) δ 181.2, 161.1, 155.2, 131.5, 130.7, 123.2, 12.6, 109.5, 56.5, 46.9, 22.4. LC-MS (ESI) *m*/*z* found: 253 [M+H]$^+$; retention time: 3.96 minutes. HRMS-ESI [M+H]$^+$ calculated for $C_{12}H_{14}ClN_2O_2^+$: 253.0666, found: 253.0736.

5-(3-chloro-4-methoxyphenyl)-4,4-dimethyl-2,4-dihydro-3H-pyrazol-3-one (6t)

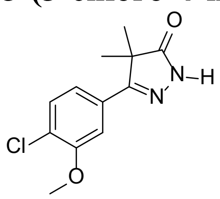

Prepared according to general method I and II using 2.0 g (10.7 mmol) of 4-chloro-3-methoxybenzoic acid. The formed precipitate was filtered off dried at 40°C *in vacuo.* The obtained solids were recrystallized from MeOH to give 1.2 g (4.8 mmol, 45%) of the title compound as a white solid. ^{1}H NMR (600 MHz, DMSO) δ 7.80 (d, J = 2.2 Hz, 1H), 7.73 (dd, J = 8.7, 2.2 Hz, 1H), 7.20 (d, J = 8.7 Hz, 1H), 3.90 (s, 3H), 1.34 (s, 6H). ^{13}C NMR (151 MHz, DMSO) δ 180.6, 160.3, 155.5, 126.9, 126.2, 124.4, 121.8, 112.9, 56.3, 46.4, 21.8. LC-MS (ESI) *m*/*z* found: 253 [M+H]$^+$; retention time: 3.79 minutes. HRMS-ESI [M+H]$^+$ calculated for $C_{12}H_{14}ClN_2O_2^+$: 253.0738, found: 253.0732.

5-(4-bromo-3-methoxyphenyl)-4,4-dimethyl-2,4-dihydro-3H-pyrazol-3-one (6u)

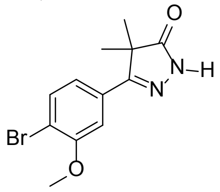

Prepared according to general method I and II using 2.0 g (8.6 mmol) of 4-bromo-3-methoxybenzoic acid. The formed precipitate was filtered off dried at 40°C *in vacuo.* The obtained solids were recrystallized from MeOH to give 1.9 g (6.4 mmol, 76%) of the title compound as a white solid. ^{1}H NMR (600 MHz, $CDCl_3$) δ 9.14 (s, 1H), 7.58 (d, J = 8.2 Hz, 1H), 7.43 (d, J = 1.9 Hz, 1H), 7.15 (dd, J = 8.2, 2.0 Hz, 1H), 3.95 (s, 3H), 1.51 (s, 6H). ^{13}C NMR (151 MHz, $CDCl_3$) δ 181.2, 162.6, 156.3, 133.5, 131.4, 12.4, 114.2, 109.1, 56.3, 47.2, 22.5. LC-MS (ESI) *m*/*z* found: 297 [M+H]$^+$; retention time: 4.07 minutes.

3-(3,4-dimethoxyphenyl)-4,4-dimethyl-1H-pyrazol-5(4H)-one (6v)

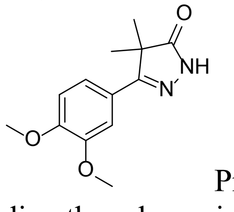

Prepared according to general method I and II using 2.0 g (11.0 mmol) of 3,4-dimethoxybenzoic acid. Formed little precipitate, the crude was purified over SiO_2 using a gradient from 20% EtOAc in n-heptane towards 60% EtOAc to give 1.50 g (6.06 mmol, 55%) of the title compound as a white solid. ^{1}H NMR (600 MHz, DMSO-d_6) δ 11.41 (s, 1H), 7.36 (s, 1H), 7.32 (d, J = 8.4 Hz, 1H), 6.99 (d, J = 8.4 Hz, 1H), 3.80 (s, 6H), 1.36 (s, 6H). ^{13}C NMR (151 MHz, DMSO) δ 180.7, 161.4, 150.3, 149.0, 123.6, 122.2, 111.4, 108.3, 55.5, 55.4, 46.4, 22.2. LC-MS (ESI) *m*/*z* found: 249 [M+H]$^+$; retention time: 3.15 minutes. HRMS-ESI [M+H]$^+$ calculated for $C_{13}H_{17}N_2O_3^+$: 249.1161, found: 249.1234.

3-(3,4-difluorophenyl)-4,4-dimethyl-1H-pyrazol-5(4H)-one (6w)

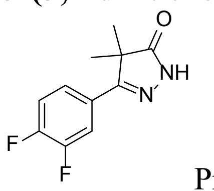

Prepared according to general method I and II using 2.0 g (12.7 mmol) of 3,4-difluorobenzoic acid. Formed little precipitate, the crude was purified over SiO_2 using a gradient from 20% EtOAc in n-heptane towards 60% EtOAc to give 1.79 g (8.0 mmol mmol, 60%) of the title compound as a yellowish solid. ^{1}H NMR (600 MHz, DMSO-d_6) δ 11.66 (s, 1H), 7.83 – 7.76 (m, 1H), 7.66 (d, J = 6.8 Hz, 1H), 7.51 (q, J = 9.0 Hz, 1H), 1.36 (s, 6H). ^{13}C NMR (151 MHz, DMSO) δ 180.6, 159.8, 151.1, 151.0, 150.6, 150.5, 149.4, 149.3, 148.9, 148.9, 128.5, 128.5, 128.5, 128.5, 123.1, 123.1, 123.1, 123.1, 118.2, 118.1, 114.7, 114.6, 46.4, 21.6. LC-MS (ESI) m/z found: 225 $[M+H]^+$; retention time: 3.78 minutes. HRMS-ESI $[M+H]^+$ calculated for $C_{11}H_{11}F_2N_2O^+$: 225.0761, found: 225.0834.

1,1'-methylenebis(3-(3-bromophenyl)-4,4-dimethyl-1H-pyrazol-5(4H)-one) (7a)

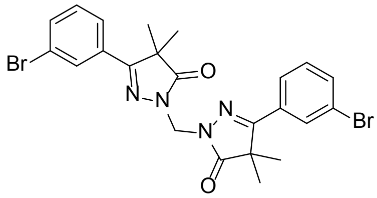

Prepared according to general method III using 200 mg (0.75 mmol) of pyrazolone **6a**. The white solid obtained was recrystallized from MeOH to give 28 mg (0.36 mmol, 97%) of the title compound as a white solid. ^{1}H NMR (600 MHz, $CDCl_3$) δ 7.91 (s, 2H), 7.68 (d, J = 7.9 Hz, 2H), 7.52 (d, J = 8.0 Hz, 2H), 7.32 – 7.22 (m, 2H), 5.70 (s, 2H), 1.51 (s, 12H). ^{13}C NMR (151 MHz, $CDCl_3$) δ 179.1, 161.4, 133.2, 132.7, 130.4, 129.4, 124.9, 123.2, 52.0, 48.5, 22.7. LC-MS (ESI) m/z found: 545 $[M+H]^+$; retention time: 5.60 minutes. HRMS-ESI $[M+H]^+$ calculated for $C_{23}H_{23}Br_2N_4O_2^+$: 545.0110, found: 545.0182.

2,2'-methylenebis(5-(2-bromophenyl)-4,4-dimethyl-2,4-dihydro-3H-pyrazol-3-one) (7b)

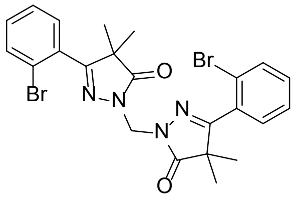

Prepared according to general method III using 200 mg (0.75 mmol) of pyrazolone **6b**. The white solid obtained was recrystallized from MeOH to give 175 mg (0.32 mmol, 86%) of the title compound as a white solid. ^{1}H NMR (600 MHz, $CDCl_3$) δ 7.67 – 7.62 (m, 2H), 7.35 (td, J = 1.0, 7.5, 8.5 Hz, 2H), 7.29 (td, J = 1.6, 8.0, 8.0 Hz, 2H), 7.20 (dd, J= 1.4, 7.6 Hz, 2H), 5.73 (s, 2H), 1.33 (s, 12H). ^{13}C NMR (151 MHz, $CDCl_3$) δ 177.8, 163.6, 133.5, 132.4, 130.9, 130.3, 127.0, 123.5, 51.3, 50.6, 21.2. LC-MS (ESI) m/z found: 545 $[M+H]^+$; retention time: 5.08 minutes. HRMS-ESI $[M+H]^+$ calculated for $C_{23}H_{23}Br_2N_4O_2^+$: 545.0110, found: 545.0182.

2,2'-methylenebis(4,4-dimethyl-5-phenyl-2,4-dihydro-3H-pyrazol-3-one) (7c)

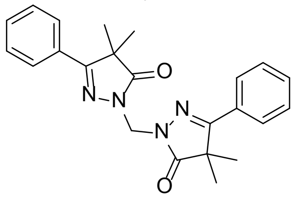

Prepared according to general method III using 200 mg (1.06 mmol) of pyrazolone **6p**. The white solid obtained was recrystallized from MeOH to give 160 mg (0.41 mmol, 78%) of the title compound as a white solid. ^{1}H NMR (300 MHz, $CDCl_3$) δ 7.80 (dd, *J* = 3.0, 6.7 Hz, 4H), 7.43 – 7.36 (m, 6H), 5.73 (s, 2H), 1.55 (s, 12H). ^{13}C NMR (151 MHz, DMSO) δ 174.5, 157.8, 125.9, 125.4, 124.0, 121.6, 47.0, 43.7, 17.9. LC-MS (ESI) *m*/*z* found: 389 $[M+H]^+$; retention time: 4.86 minutes. HRMS-ESI $[M+H]^+$ calculated for $C_{23}H_{25}N_4O_2^+$: 389.1895, found: 389.272.

1,1'-methylenebis(3-(4-chlorophenyl)-4,4-dimethyl-1H-pyrazol-5(4H)-one) (7d)

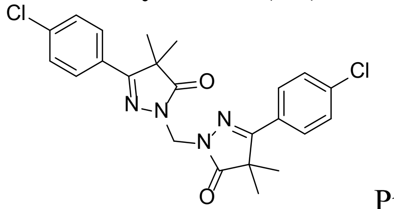

Prepared according to general method III using 200 mg (0.90 mmol) of pyrazolone **6c**. The white solid obtained was recrystallized from MeOH to give 115 mg (0.25 mmol, 56%) of the title compound as a white solid. ^{1}H NMR (300 MHz, $CDCl_3$) δ 7.71 (d, *J* = 8.6 Hz, 4H), 7.36 (d, *J* = 8.6 Hz, 4H), 5.69 (s, 2H), 1.49 (s, 12H). ^{13}C NMR (151 MHz, $CDCl_3$) δ 179.0, 161.6, 136.3, 129.1, 129.0, 127.6, 51.8, 48.3, 22.6. LC-MS (ESI) *m*/*z* found: 457 $[M+H]^+$; retention time: 5.51 minutes. HRMS-ESI $[M+H]^+$ calculated for $C_{23}H_{23}Cl_2N_4O_2^+$: 457.1120, found: 457.123.

2,2'-methylenebis(5-(3-chlorophenyl)-4,4-dimethyl-2,4-dihydro-3H-pyrazol-3-one) (7e)

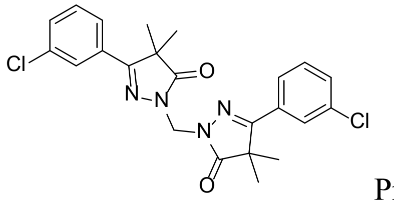

Prepared according to general method III using 200 mg (0.90 mmol) of pyrazolone **6d**. The white solid obtained was recrystallized from MeOH to give 148 mg (0.32 mmol, 72%) of the title compound as a white solid. ^{1}H NMR (600 MHz, $CDCl_3$) δ 7.76 (t, *J* = 1.7, 1.7 Hz, 2H), 7.63 (d, *J* = 7.8 Hz, 2H), 7.37 (d, *J* = 8.1 Hz, 2H), 7.32 (t, *J* = 7.9, 7.9 Hz, 2H), 5.70 (s, 2H), 1.51 (s, 12H). ^{13}C NMR (151 MHz, $CDCl_3$) δ 179.3, 161.7, 135.3, 132.6, 130.5, 130.4, 126.7, 124.7, 77.2, 52.2, 48.7, 22.9. LC-MS (ESI) *m*/*z* found: 457 $[M+H]^+$; retention time: 5.54 minutes. HRMS-ESI $[M+H]^+$ calculated for $C_{23}H_{23}Cl_2N_4O_2^+$: 457.1120, found: 457.123.

2,2'-methylenebis(5-(2-chlorophenyl)-4,4-dimethyl-2,4-dihydro-3H-pyrazol-3-one) (7f)

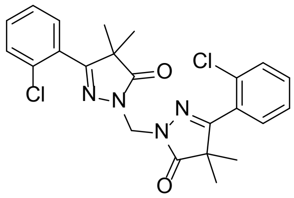

Prepared according to general method III using 200 mg (0.90 mmol) of pyrazolone **6e**. The white solid obtained was recrystallized from MeOH to give 124 mg (0.271 mmol, 60%) of the title compound as a white solid. ^{1}H NMR (600 MHz, $CDCl_3$) δ 7.47 – 7.44 (m, 2H), 7.37 (td, *J* = 1.6, 7.8, 8.0 Hz, 2H), 7.31 (td, *J* = 1.1, 7.5, 7.5 Hz, 2H), 7.21 (dd, *J*= 1.5, 7.6 Hz, 2H), 5.72 (s, 2H), 1.32 (s, 12H). ^{13}C NMR (151 MHz, $CDCl_3$) δ 177.9, 162.9, 133.9, 130.7, 130.5, 130.4, 130.3, 126.5, 51.3, 50.6, 21.1. LC-MS (ESI) *m*/*z* found: 457 $[M+H]^+$; retention time: 4.98 minutes. HRMS-ESI $[M+H]^+$ calculated for $C_{23}H_{23}Cl_2N_4O_2^+$: 457.1120, found: 457.123.

1,1'-methylenebis(3-(4-fluorophenyl)-4,4-dimethyl-1H-pyrazol-5(4H)-one) (7g)

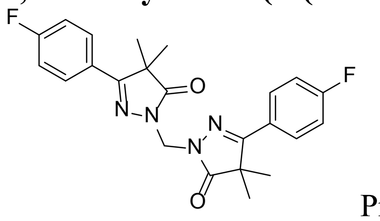

Prepared according to general method III using 200 mg (0.97 mmol) of pyrazolone **6f**. The white solid obtained was recrystallized from MeOH to give 136 mg (0.32 mmol, 66%) of the title compound as a white solid. ^{1}H NMR (600 MHz, $CDCl_3$) δ 7.77 (dd, *J* = 5.6, 8.0 Hz, 4H), 7.08 (t, *J* = 8.4, 8.4 Hz, 4H), 5.68 (s, 2H), 1.50 (s, 12H). ^{13}C NMR (151 MHz, $CDCl_3$) δ 179.0, 164.6, 162.9, 161.6, 128.4, 128.3, 126.9, 126.8, 116.0, 115.9, 51.7, 48.3, 22.6. LC-MS (ESI) *m*/*z* found: 425 $[M+H]^+$; retention time: 4.93 minutes. HRMS-ESI $[M+H]^+$ calculated for $C_{23}H_{23}F_2N_4O_2^+$: 425.1711, found: 425.1784.

1,1'-methylenebis(3-(3-fluorophenyl)-4,4-dimethyl-1H-pyrazol-5(4H)-one) (7h)

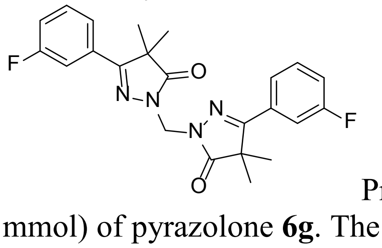

Prepared according to general method III using 200 mg (0.97 mmol) of pyrazolone **6g**. The white solid obtained was recrystallized from MeOH to give 122 mg (0.29 mmol, 59%) of the title compound as a white solid. ^{1}H NMR (600 MHz, $CDCl_3$) δ 7.53 (d, *J* = 7.9 Hz, 2H), 7.49 (d, *J* = 10.1 Hz, 2H), 7.36 (q, *J* = 7.9, 8.0, 8.0 Hz, 2H), 7.09 (td, *J* = 2.2, 8.2, 8.3 Hz, 2H), 5.70 (s, 2H), 1.51 (s, 12H). ^{13}C NMR (151 MHz, $CDCl_3$) δ 179.0, 163.7, 162.1, 161.5, 161.5, 132.6, 132.6, 130.4, 130.3, 122.0, 122.0, 117.3, 117.1, 113.3, 113.1, 51.8, 48.3, 22.6. LC-MS (ESI) *m*/*z* found: 425 $[M+H]^+$; retention time: 4.98 minutes. HRMS-ESI $[M+H]^+$ calculated for $C_{23}H_{23}F_2N_4O_2^+$: 425.1711, found: 425.1784

2,2'-methylenebis(5-(2-fluorophenyl)-4,4-dimethyl-2,4-dihydro-3H-pyrazol-3-one) (7i)

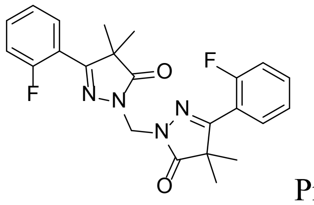

Prepared according to general method III using 200 mg (0.97 mmol) of pyrazolone **6h**. The white solid obtained was recrystallized from MeOH to give 170 mg (0.40 mmol, 83%) of the title compound as a white solid. ^{1}H NMR (600 MHz, $CDCl_3$) δ 7.63 (td, *J* = 1.6, 7.6, 7.6 Hz, 2H), 7.42 – 7.37 (m, 2H), 7.17 (td, *J* = 1.0, 7.8, 7.8 Hz, 2H), 7.13 – 7.09 (m, 2H), 5.71 (s, 2H), 1.39 (s, 12H). ^{13}C NMR (151 MHz, $CDCl_3$) δ 179.0, 160.9, 160.8, 160.8, 159.2, 131.8, 131.7, 130.0, 130.0, 124.3, 124.3, 120.1, 119.0, 116.7, 116.5, 51.7, 49.7, 21.3, 21.2. LC-MS (ESI) *m/z* found: 425 [M+H]$^+$; retention time: 4.86 minutes. HRMS-ESI [M+H]$^+$ calculated for $C_{23}H_{23}F_2N_4O_2^+$: 425.1711, found: 425.1784.

1,1'-methylenebis(3-(4-methoxyphenyl)-4,4-dimethyl-1H-pyrazol-5(4H)-one) (7j)

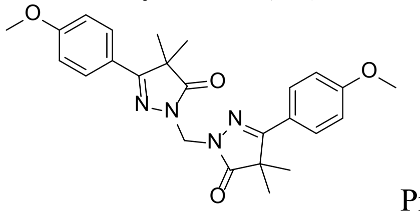

Prepared according to general method III using 200 mg (0.92 mmol) of pyrazolone **6i**. The white solid obtained was recrystallized from MeOH to give 170 mg (0.38 mmol, 83%) of the title compound as a white solid. ^{1}H NMR (600 MHz, $CDCl_3$) δ 7.72 (d, *J* = 8.8 Hz, 4H), 6.89 (d, *J* = 8.8 Hz, 4H), 5.67 (s, 2H), 3.82 (s, 6H), 1.50 (s, 12H). ^{13}C NMR (151 MHz, $CDCl_3$) δ (ppm) 179.6, 162.7, 161.4, 128.3, 123.7, 114.5, 55.7, 52.0, 48.7, 23.1. LC-MS (ESI) *m/z* found: 449 [M+H]$^+$; retention time: 4.72 minutes. HRMS-ESI [M+H]$^+$ calculated for $C_{25}H_{29}N_4O_4^+$: 449.2111, found: 449.2183.

1,1'-methylenebis(3-(3-methoxyphenyl)-4,4-dimethyl-1H-pyrazol-5(4H)-one) (7k)

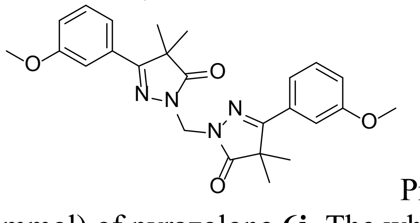

Prepared according to general method III using 150 mg (0.69 mmol) of pyrazolone **6j**. The white solid obtained was recrystallized from MeOH to give 144 mg (0.32 mmol, 93%) of the title compound as a white solid. ^{1}H NMR (600 MHz, $CDCl_3$) δ 7.34 – 7.27 (m, 6H), 6.94 (d, *J* = 9.0 Hz, 2H), 5.71 (s, 2H), 3.81 (s, 6H), 1.51 (s, 12H). ^{13}C NMR (151 MHz, $CDCl_3$) δ 179.3, 162.4, 159.8, 131.8, 129.7, 118.8, 116.2, 111.4, 55.3, 51.7, 48.5, 22.7. LC-MS (ESI) *m/z* found: 449 [M+H]$^+$; retention time: 4.84 minutes. HRMS-ESI [M+H]$^+$ calculated for $C_{25}H_{29}N_4O_4^+$: 449.2111, found: 449.2183.

2,2'-methylenebis(5-(2-methoxyphenyl)-4,4-dimethyl-2,4-dihydro-3H-pyrazol-3-one) (7l)

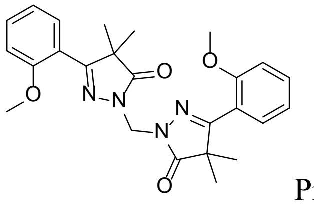

Prepared according to general method III using 200 mg (0.92 mmol) of pyrazolone **6k**. The white solid obtained was recrystallized from MeOH to give 140 mg (0.31 mmol, 68%) of the title compound as a white solid. ^{1}H NMR (600 MHz, $CDCl_3$) δ 7.38 (td, *J* = 1.7, 8.4, 8.4 Hz, 2H), 7.27 (dd, *J* = 1.6, 7.6 Hz, 2H), 6.98 – 6.94 (m, 2H), 6.93 (d, *J* = 8.3 Hz, 2H), 5.69 (s, 2H), 3.76 (s, 6H), 1.30 (s, 12H). ^{13}C NMR (151 MHz, $CDCl_3$) δ 179.2, 164.2, 157.7, 131.1, 130.6, 120.8, 120.4, 111.3, 55.2, 51.8, 50.5, 21.5. LC-MS (ESI) *m*/*z* found: 449 $[M+H]^+$; retention time: 4.69 minutes. HRMS-ESI $[M+H]^+$ calculated for $C_{25}H_{29}N_4O_4^+$: 449.2111, found: 449.2183.

2,2'-methylenebis(5-(4-(dimethylamino)phenyl)-4,4-dimethyl-2,4-dihydro-3H-pyrazol-3-one) (7m)

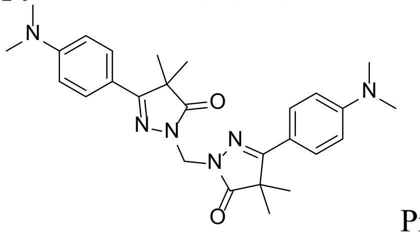

Prepared according to general method III using 200 mg (0.87 mmol) of pyrazolone **6l**. The white solid obtained was recrystallized from MeOH to give 96 mg (0.20 mmol, 47%) of the title compound as a white solid. ^{1}H NMR (300 MHz, $CDCl_3$) δ 7.70 (d, *J* = 9.0 Hz, 4H), 6.74 (d, *J* = 9.0 Hz, 4H), 5.68 (s, 2H), 3.02 (s, 12H), 1.52 (s, 12H). ^{13}C NMR (151 MHz, $CDCl_3$) δ 180.1, 163.1, 151.3, 128.4, 122.3, 113.6, 52.3, 49.0, 41.7, 23.6. LC-MS (ESI) *m*/*z* found: 475 $[M+H]^+$; retention time: 2.06 minutes. HRMS-ESI $[M+H]^+$ calculated for $C_{27}H_{35}N_6O_2^+$: 475.2743, found: 475.2816.

2,2'-methylenebis(5-(3-(dimethylamino)phenyl)-4,4-dimethyl-2,4-dihydro-3H-pyrazol-3-one) (7n)

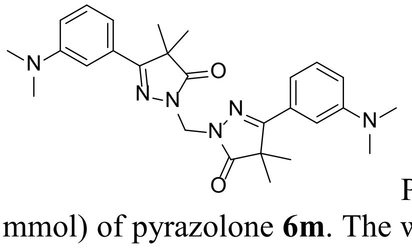

Prepared according to general method III using 200 mg (0.87 mmol) of pyrazolone **6m**. The white solid obtained was recrystallized from MeOH to give 93 mg (0.20 mmol, 45%) of the title compound as a white solid. ^{1}H NMR (300 MHz, $CDCl_3$) δ 7.21 (d, *J* = 8.0 Hz, 1H), 7.16 (s, 2H), 7.05 (d, *J* = 7.6 Hz, 2H), 6.78 (d, *J* = 7.8 Hz, 2H), 5.71 (s, 2H), 2.94 (s, 12H), 1.51 (s, 12H). ^{13}C NMR (151 MHz, $CDCl_3$) δ 179.3, 163.1, 150.2, 131.0, 129.0, 114.6, 114.4, 109.6, 51.4, 48.4, 40.3, 22.6. LC-MS (ESI) *m*/*z* found: 475 $[M+H]^+$; retention time: 4.60 minutes. HRMS-ESI $[M+H]^+$ calculated for $C_{27}H_{35}N_6O_2^+$: 475.2743, found: 475.2816.

4,4'-(methylenebis(4,4-dimethyl-5-oxo-4,5-dihydro-1H-pyrazole-1,3-diyl))dibenzonitrile (7o)

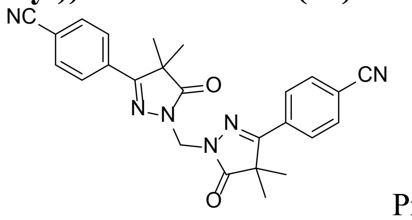

Prepared according to general method III using 200 mg (0.94 mmol) of pyrazolone **6n**. The solid obtained was recrystallized from MeOH to give 83 mg (0.2 mmol, 40%) of the title compound as a white solid. ^{1}H NMR (600 MHz, $CDCl_3$) δ 7.88 (d, *J* = 8.6 Hz, 1H), 7.68 (d, *J* = 8.6 Hz, 1H), 5.73 (s, 0H), 1.52 (s, 3H). ^{13}C NMR (151 MHz, $CDCl_3$) δ 179.0, 161.1, 134.8, 132.9, 127.0, 118.5, 113.9, 77.2, 52.3, 48.5, 22.9. LC-MS (ESI) *m*/*z* found: 439 $[M+H]^+$; retention time: 4.52 minutes. HRMS-ESI $[M+H]^+$ calculated for $C_{25}H_{23}N_6O_2^+$: 439.1804, found: 439.1877.

3,3'-(methylenebis(4,4-dimethyl-5-oxo-4,5-dihydro-1H-pyrazole-1,3-diyl))dibenzonitrile (7p)

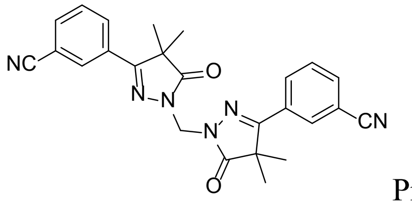

Prepared according to general method III using 200 mg (0.94 mmol) of pyrazolone **6o**. The white solid obtained was recrystallized from MeOH to give 132 mg (0.30 mmol, 64%) of the title compound as a yellow solid. ^{1}H NMR (600 MHz, $CDCl_3$) δ 8.06 (s, 2H), 8.04 (d, *J* = 8.1 Hz, 2H), 7.71 (d, *J* = 7.7 Hz, 2H), 7.56 (t, *J* = 7.9, 7.9 Hz, 2H), 5.75 (s, 2H), 1.55 (s, 12H). ^{13}C NMR (151 MHz, $CDCl_3$) δ 178.9, 160.8, 133.6, 132.1, 130.6, 130.1, 130.1, 118.4, 113.7, 77.2, 52.3, 48.5, 22.8. LC-MS (ESI) *m*/*z* found: 439 $[M+H]^+$; retention time: 4.48 minutes. HRMS-ESI $[M+H]^+$ calculated for $C_{25}H_{23}N_6O_2^+$: 439.1804, found: 439.1877.

2,2'-methylenebis(5-(3-fluoro-4-methoxyphenyl)-4,4-dimethyl-2,4-dihydro-3H-pyrazol-3-one) (7q)

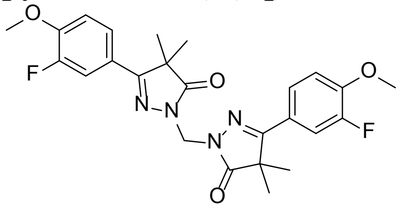

Prepared according to general method III using 200 mg (0.85 mmol) of pyrazolone **6q**. The white solid obtained was recrystallized from MeOH to give 74 mg (0.15 mmol, 36 %) of the title compound as a white solid. ^{1}H NMR (600 MHz, $CDCl_3$) δ 7.57 (d, 1H), 7.53 (dd, *J* = 12.6, 2.1 Hz, 1H), 7.21 (t, *J* = 8.8 Hz, 1H), 5.55 (s, 1H), 3.87 (s, 3H), 1.38 (s, 6H). ^{13}C NMR (151 MHz, $CDCl_3$) δ 178.4, 160.9 (d, *J* = 2 Hz), 151.4 (d, *J* = 245 Hz), 148.9 (d, *J* = 11 Hz), 123.3, 122.9 (d, *J* = 7 Hz), 114.0, 113.1 (d, *J* = 20 Hz), 56.2, 51.6, 47.6, 22.0. LC-MS (ESI) *m*/*z* found: 485 $[M+H]^+$; retention time: 4.81 minutes. HRMS-ESI $[M+H]^+$ calculated for $C_{25}H_{27}F_2N_4O_4^+$: 485.295, found: 485.296.

2,2'-methylenebis(5-(3-chloro-4-methoxyphenyl)-4,4-dimethyl-2,4-dihydro-3H-pyrazol-3-one) (7r)

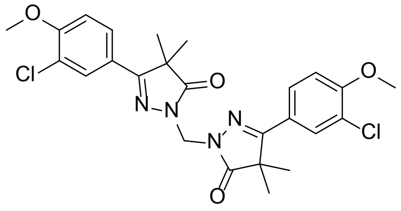
Prepared according to general method III using 200 mg (1.06 mmol) of pyrazolone **6r**. The white solid obtained was recrystallized twice from MeOH to give 30 mg (0.06 mmol, 14 %) of the title compound as a white solid. ^{1}H NMR (600 MHz, DMSO-d6) δ 7.76 (d, *J* = 2.2 Hz, 2H), 7.72 (dd, *J* = 8.7, 2.3 Hz, 2H), 7.20 (d, *J* = 8.7 Hz, 2H), 5.56 (s, 2H), 3.90 (s, 6H), 1.39 (s, 12H). ^{13}C NMR (151 MHz, DMSO-d6) δ 178.4, 160.7, 155.9, 127.0, 126.6, 123.4, 121.9, 113.0, 56.4, 51.7, 47.6, 39.5, 21.9. LC-MS (ESI) *m/z* found: 517 $[M+H]^+$; retention time: 5.18 minutes.

2,2'-methylenebis(5-(4-chloro-3-methoxyphenyl)-4,4-dimethyl-2,4-dihydro-3H-pyrazol-3-one) (7t)

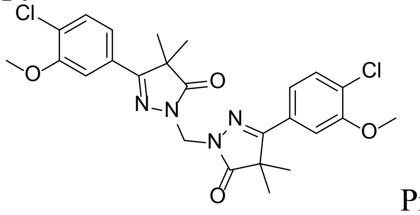
Prepared according to general method III using 200 mg (0.79 mmol) of pyrazolone **6t**. The white solid obtained was recrystallized from MeOH to give 98 mg (0.20 mmol, 48%) of the title compound as a white solid. ^{1}H NMR (600 MHz, $CDCl_3$) δ 7.42 (d, *J* = 1.7 Hz, 2H), 7.36 (d, *J* = 8.2 Hz, 2H), 7.2 (dd, *J* = 1.9, 8.3 Hz, 2H), 5.71 (s, 2H), 3.90 (s, 6H), 1.51 (s, 12H). ^{13}C NMR (151 MHz, $CDCl_3$) δ 179.1, 161.7, 155.4, 130.3, 130.2, 125.1, 122.2, 109.3, 56.2, 51.8, 48.3, 22.8. LC-MS (ESI) *m/z* found: 517 $[M+H]^+$; retention time: 5.40 minutes. HRMS-ESI $[M+H]^+$ calculated for $C_{25}H_{27}Cl_2N_4O_4^+$: 517.1331, found: 517.1404.

2,2'-methylenebis(5-(4-bromo-3-methoxyphenyl)-4,4-dimethyl-2,4-dihydro-3H-pyrazol-3-one) (7u)

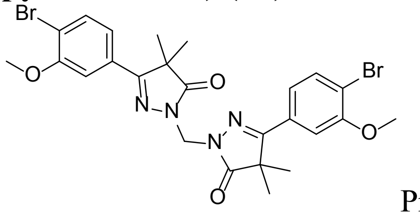
Prepared according to general method III using 200 mg (0.67 mmol) of pyrazolone **6u**. The white solid obtained was recrystalized from MeOH to give 140 mg (0.47 mmol, 70%) of the title compound as a white solid. ^{1}H NMR (600 MHz, $CDCl_3$) δ 7.47 (d, J = 8.2 Hz, 1H), 7.32 (d, J = 1.8 Hz, 1H), 7.06 (dd, J = 8.2, 1.9 Hz, 1H), 5.64 (s, 1H), 3.83 (s, 3H), 1.44 (s, 6H). ^{13}C NMR (151 MHz, $CDCl_3$) δ 179.1, 161.8, 156.3, 133.4, 130.9, 12.6, 114.4, 109.1, 56.2, 51.8, 48.3, 22.8. LC-MS (ESI) *m/z* found: 607 $[M+H]^+$; retention time: 5.53 minutes.

1,1'-methylenebis(3-(3,4-dimethoxyphenyl)-4,4-dimethyl-1H-pyrazol-5(4H)-one) (7v)

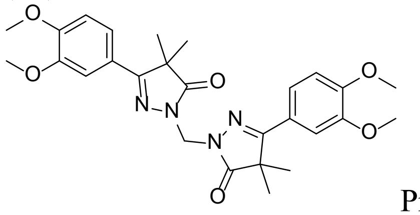

Prepared according to general method III using 200 mg (0.81 mmol) of pyrazolone **6t**. The white solid obtained was recrystallized from MeOH to give 120 mg (0.24 mmol, 59%) of the title compound as a white solid. ^{1}H NMR (600 MHz, $CDCl_3$) δ 7.41 (d, *J* = 1.9 Hz, 2H), 7.23 (dd, *J* = 2.0, 8.4 Hz, 2H), 6.83 (d, *J* = 8.4 Hz, 2H), 5.69 (s, 2H), 3.89 (s, 6H), 3.87 (s, 6H), 1.51 (s, 12H). ^{13}C NMR (151 MHz, $CDCl_3$) δ 179.8, 162.6, 151.3, 149.7, 123.8, 123.0, 110.8, 109.1, 56.3, 56.2, 52.1, 48.8, 23.3. LC-MS (ESI) *m*/*z* found: 509 $[M+H]^+$; retention time: 4.20 minutes. HRMS-ESI $[M+H]^+$ calculated for $C_{27}H_{33}N_4O_6^+$: 509.2322, found: 509.2395.

1,1'-methylenebis(3-(3,4-difluorophenyl)-4,4-dimethyl-1H-pyrazol-5(4H)-one) (7w)

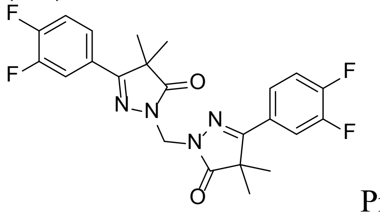

Prepared according to general method III using 200 mg (0.89 mmol) of pyrazolone **6u**. The white solid obtained was recrystallized from MeOH to give 147 mg (0.32 mmol, 72%) of the title compound as a white solid. ^{1}H NMR (600 MHz, $CDCl_3$) δ 7.68 – 7.62 (m, 2H), 7.51 (d, *J* = 8.6 Hz, 2H), 7.21 (q, *J* = 8.7, 8.7, 8.8 Hz, 2H), 5.70 (s, 2H), 1.52 (s, 12H). ^{13}C NMR (151 MHz, $CDCl_3$) δ 178.8, 160.7, 152.4, 152.3, 151.4, 151.3, 150.7, 150.6, 149.8, 149.7, 127.7, 127.6, 127.6, 127.6, 122.8, 122.7, 122.7, 122.7, 117.8, 117.7, 51.8, 48.2, 22.6. LC-MS (ESI) *m*/*z* found: 461 $[M+H]^+$; retention time: 5.13 minutes. HRMS-ESI $[M+H]^+$ calculated for $C_{23}H_{21}F_4N_4O_2^+$: 461.1522, found: 461.1595.

4,4-dimethyl-3-(thiophen-2-yl)-1H-pyrazol-5(4H)-one (10a)

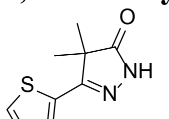

Prepared according to general method I and II using 2.0 g (15.6 mmol) of thiophene-2-carboxilic acid. The crude was purified over SiO_2 using a gradient from 30% EtOAc in n-heptane towards 60% EtOAc to give 1.95 g (10.0 mmol, 64%) of the title compound as a yellowish solid. ^{1}H NMR (600 MHz, DMSO) δ 11.47 (s, 1H), 7.65 (d, *J* = 5.1 Hz, 1H), 7.57 (d, *J* = 3.6 Hz, 1H), 7.14 (t, *J* = 4.3, 4.3 Hz, 1H), 1.36 (s, 6H). ^{13}C NMR (151 MHz, DMSO) δ 180.3, 158.4, 134.5, 128.2, 128.1, 127.2, 46.8, 22.1. LC-MS (ESI) *m*/*z* found: 25 $[M+H]^+$; retention time: 3.25 minutes. HRMS-ESI $[M+H]^+$ calculated for $C_9H_{11}N_2OS^+$: 25.0514, found: 25.0587.

4,4-dimethyl-5-(thiophen-3-yl)-2,4-dihydro-3H-pyrazol-3-one (10b)

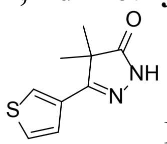

Prepared according to general method I and II using 2.0 g (15.6 mmol) of thiophene-3-carboxilic acid. The crude was purified over SiO_2 using a gradient from 30%

EtOAc in n-heptane towards 60% EtOAc to give 0.95 g (4.9 mmol, 31%) of the title compound as a yellowish solid. ^{1}H NMR (300 MHz, DMSO-d_6) δ 11.40 (s, 1H), 8.06 (dd, J = 2.7, 1.2 Hz, 1H), 7.64 (dd, J = 5.1, 2.7 Hz, 1H), 7.50 (dd, J = 5.1, 1.1 Hz, 1H), 1.34 (s, 6H). ^{13}C NMR (151 MHz, $CDCl_3$) δ 180.7, 159.3, 132.7, 127.2, 125.5, 125.2, 46.5, 39.6, 22.0. LC-MS (ESI) m/z found: 25 $[M+H]^+$; retention time: 3.26 minutes. HRMS-ESI $[M+H]^+$ calculated for $C_9H_{11}N_2OS^+$: 25.0514, found: 25.0587.

5-(furan-2-yl)-4,4-dimethyl-2,4-dihydro-3H-pyrazol-3-one (10c)

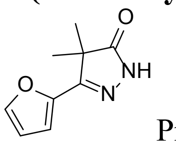

Prepared according to general method I and II using 2.0 g (17.8 mmol) of furan-2-carboxilic acid. The crude was purified over SiO_2 using a gradient from 30% EtOAc in n-heptane towards 60% EtOAc to give 0.51 g (2.9 mmol, 16%) of the title compound as a white solid. ^{1}H NMR (600 MHz, DMSO) δ 11.50 (s, 1H), 7.84 (dd, J = 0.7, 1.8 Hz, 1H), 7.03 (d, J = 3.4 Hz, 1H), 6.65 (dd, J = 1.8, 3.5 Hz, 1H), 1.32 (s, 6H). ^{13}C NMR (151 MHz, DMSO) δ 180.2, 155.0, 146.4, 145.0, 112.4, 111.6, 46.5, 22.2. LC-MS (ESI) m/z found: 179 $[M+H]^+$; retention time: 2.91 minutes. HRMS-ESI $[M+H]^+$ calculated for $C_9H_{11}N_2O_2^+$: 179.0742, found: 179.0815.

3-(furan-3-yl)-4,4-dimethyl-1H-pyrazol-5(4H)-one (10d)

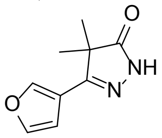

Prepared according to general method I and II using 2.0 g (17.8 mmol) of furan-3-carboxilic acid. The crude was purified over SiO_2 using a gradient from 30% EtOAc in n-heptane towards 60% EtOAc to give 0.80 g (4.49 mmol, 25%) of the title compound as a yellowish solid. ^{1}H NMR (600 MHz, DMSO-d_6) δ 11.37 (s, 1H), 8.34 (s, 1H), 7.77 (t, J = 1.5 Hz, 1H), 6.79 – 6.77 (m, 1H), 1.28 (s, 6H). ^{13}C NMR (151 MHz, DMSO) δ 180.5, 157.3, 144.3, 142.2, 117.8, 107.5, 46.3, 21.7. LC-MS (ESI) m/z found: 179 $[M+H]^+$; retention time: 2.89 minutes. HRMS-ESI $[M+H]^+$ calculated for $C_9H_{11}N_2O_2^+$: 179.0742, found: 179.0815.

4,4-dimethyl-3-(thiazol-4-yl)-1H-pyrazol-5(4H)-one (10e)

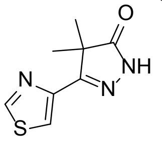

Prepared according to general method I and II using 0.85 g (6.58 mmol) of thiazole-4-carboxilic acid. The crude was purified over SiO_2 using a gradient from 30% EtOAc in n-heptane towards 60% EtOAc to give 0.7 g (3.6 mmol, 55%) of the title compound as a yellowish solid. ^{1}H NMR (600 MHz, DMSO-d_6) δ 11.50 (s, 1H), 9.22 (s, 1H), 8.20 (s, 1H), 1.36 (s, 6H). ^{13}C NMR (151 MHz, DMSO) δ 180.3, 158.3, 155.0, 148.1, 118.7, 46.8, 21.3. LC-MS (ESI) m/z found: 26 $[M+H]^+$; retention time: 2.61 minutes. HRMS-ESI $[M+H]^+$ calculated for $C_8H_{10}N_3OS^+$: 26.0466, found: 26.0539.

5-(5-bromothiophen-2-yl)-4,4-dimethyl-2,4-dihydro-3H-pyrazol-3-one (10f)

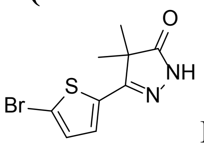

Prepared according to general method I and II using 2.0 g (9.7 mmol) of 5-bromothiophene-2-carboxilic acid. The crude was purified over SiO_2 using a gradient from

30% EtOAc in n-heptane towards 60% EtOAc to give 0.61 g (2.2 mmol, 23%) of the title compound as a yellowish solid. ^{1}H NMR (300 MHz, DMSO) δ 11.57 (s, 1H), 7.42 (dd, *J* = 0.9, 4.0 Hz, 1H), 7.27 (dd, *J* = 0.9, 3.9 Hz, 1H), 1.35 (s, 6H). ^{13}C NMR (151 MHz, $CDCl_3$) δ 180.0, 157.5, 136.3, 131.4, 127.9, 114.0, 46.6, 21.8. LC-MS (ESI) *m/z* found: 273 $[M+H]^+$; retention time: 4.00 minutes. HRMS-ESI $[M+H]^+$ calculated for $C_9H_{11}N_2OS^+$: 272.962, found: 272.9692.

4,4-dimethyl-2,4-dihydro-3H-pyrazol-3-one (10g)

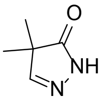

Prepared according to general method I and II using 2.0 g (17.7 mmol) of oxazole-5-carboxilic acid. The crude was purified over SiO_2 using a gradient from 30% EtOAc in n-heptane towards 60% EtOAc to give 0.47 g (4.2 mmol, 24%) of the title compound as a pale-yellow solid. ^{1}H NMR (600 MHz, DMSO) δ 11.15 (s, 1H), 7.37 (s, 1H), 1.10 (s, 6H). ^{13}C NMR (151 MHz, DMSO) δ 180.3, 157.5, 46.0, 20.0. LC-MS (ESI) *m/z* found: no mass observed $[M+H]^+$; retention time: 1.71 minutes. HRMS-ESI $[M+H]^+$ calculated for $C_5H_9N_2O^+$: 113.0637, found: 113.0709.

1,1'-methylenebis(4,4-dimethyl-3-(thiophen-2-yl)-1H-pyrazol-5(4H)-one) (11a)

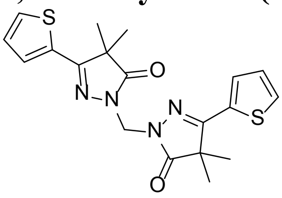

Prepared according to general method III using 200 mg (1.03 mmol) of pyrazolone **10a**. The white solid obtained was recrystallized from MeOH to give 108 mg (0.27 mmol, 52%) of the title compound as a white solid. ^{1}H NMR (600 MHz, $CDCl_3$) δ 7.38 – 7.34 (m, 4H), 7.05 (t, *J* = 4.3, 4.3 Hz, 2H), 5.64 (s, 2H), 1.51 (s, 12H). ^{13}C NMR (151 MHz, $CDCl_3$) δ 178.9, 159.2, 134.3, 128.2, 127.7, 127.1, 51.8, 48.8, 22.9. LC-MS (ESI) *m/z* found: 401 $[M+H]^+$; retention time: 4.55 minutes. HRMS-ESI $[M+H]^+$ calculated for $C_2H_{21}N_4O_2S_2^+$: 401.1028, found: 401.1100.

2,2'-methylenebis(4,4-dimethyl-5-(thiophen-3-yl)-2,4-dihydro-3H-pyrazol-3-one) (11b)

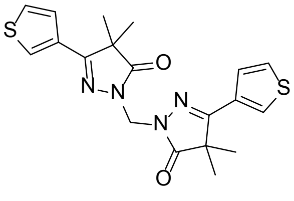

Prepared according to general method III using 200 mg (1.03 mmol) of pyrazolone **10b**. The white solid obtained was recrystallized from MeOH to give 134 mg (0.34 mmol, 65%) of the title compound as a white solid. ^{1}H NMR (600 MHz, $CDCl_3$) δ 7.59 (dd, *J* = 1.1, 2.7 Hz, 2H), 7.51 (dd, *J* = 1.0, 5.1 Hz, 2H), 7.32 (dd, *J* = 2.8, 5.1 Hz, 2H), 5.65 (s, 2H), 1.48 (s, 12H). ^{13}C NMR (151 MHz, $CDCl_3$) δ 178.9, 159.6, 132.4, 126.3, 125.8, 124.5, 51.6, 48.3, 22.7. LC-MS (ESI) *m/z* found: 401 $[M+H]^+$; retention time: 4.58 minutes. HRMS-ESI $[M+H]^+$ calculated for $C_2H_{21}N_4O_2S_2^+$: 401.1028, found: 401.1100

2,2'-methylenebis(5-(furan-2-yl)-4,4-dimethyl-2,4-dihydro-3H-pyrazol-3-one) (11c)

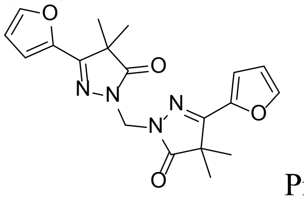

Prepared according to general method III using 200 mg (1.12 mmol) of pyrazolone **10c**. The white solid obtained was recrystallized from MeOH to give 142 mg (0.39 mmol, 69%) of the title compound as a white solid. ^{1}H NMR (600 MHz, $CDCl_3$) δ 7.50 (d, *J* = 1.2 Hz, 2H), 6.82 (d, *J* = 3.4 Hz, 2H), 6.48 (dd, *J* = 1.7, 3.5 Hz, 2H), 5.64 (s, 2H), 1.47 (s, 12H). ^{13}C NMR (151 MHz, $CDCl_3$) δ 178.2, 155.6, 146.6, 144.1, 111.7, 111.2, 52.1, 48.1, 22.1. LC-MS (ESI) *m/z* found: 369 $[M+H]^+$; retention time: 4.13 minutes. HRMS-ESI $[M+H]^+$ calculated for $C_2H_{21}N_4O_4^+$: 369.1485, found: 369.1557.

2,2'-methylenebis(5-(furan-3-yl)-4,4-dimethyl-2,4-dihydro-3H-pyrazol-3-one) (11d)

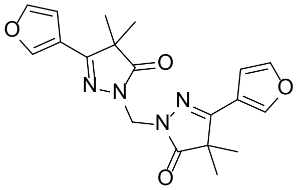

Prepared according to general method III using 200 mg (1.12 mmol) of pyrazolone **10d**. The white solid obtained was recrystallized from MeOH to give 110 mg (0.30 mmol, 53%) of the title compound as a white solid. ^{1}H NMR (600 MHz, $CDCl_3$) δ 7.74 (s, 2H), 7.42 (t, *J* = 1.5, 1.5 Hz, 2H), 6.75 (d, *J* = 1.3 Hz, 2H), 5.62 (s, 2H), 1.41 (s, 12H). ^{13}C NMR (151 MHz, $CDCl_3$) δ 179.2, 158.3, 144.1, 141.7, 118.2, 108.3, 77.2, 52.0, 48.5, 23.0. LC-MS (ESI) *m/z* found: 369 $[M+H]^+$; retention time: 4.06 minutes. HRMS-ESI $[M+H]^+$ calculated for $C_2H_{21}N_4O_4^+$: 369.1485, found: 369.1557.

2,2'-methylenebis(4,4-dimethyl-5-(thiazol-4-yl)-2,4-dihydro-3H-pyrazol-3-one) (11e)

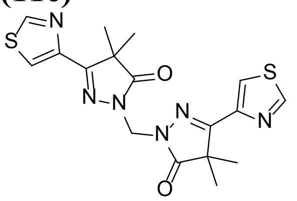

Prepared according to general method II using 150 mg (0.77 mmol) of pyrazolone **10e**. The white solid obtained was recrystallized from MeOH to give 85 mg (0.211 mmol, 55%) of the title compound as a white solid. ^{1}H NMR (600 MHz, $CDCl_3$) δ 8.81 (d, *J* = 2.0 Hz, 2H), 7.91 (d, *J* = 2.0 Hz, 2H), 5.68 (s, 2H), 1.54 (s, 12H). ^{13}C NMR (151 MHz, $CDCl_3$) δ 179.3, 159.6, 153.1, 148.8, 118.8, 52.1, 49.3, 22.0. LC-MS (ESI) *m/z* found: 403 $[M+H]^+$; retention time: 3.70 minutes. HRMS-ESI $[M+H]^+$ calculated for $C_{17}H_2N_6O_2S_2^+$: 403.0933, found: 403.1055.

2,2'-methylenebis(5-(5-bromothiophen-2-yl)-4,4-dimethyl-2,4-dihydro-3H-pyrazol-3-one) (11f)

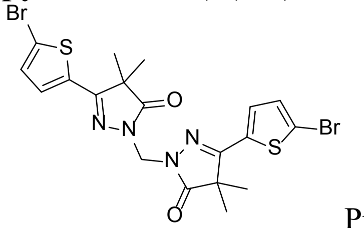

Prepared according to general method III using 150 mg (0.55 mmol) of pyrazolone **10f**. The white solid obtained was recrystallized from MeOH to give 58 mg (0.10 mmol, 38%) of the title compound as a white solid. ^{1}H NMR (600 MHz, $CDCl_3$) δ 7.08 (d, *J* = 4.0 Hz, 2H), 7.00 (d, *J* = 4.0 Hz, 2H), 5.59 (s, 2H), 1.48 (s, 12H). ^{13}C NMR (151 MHz, $CDCl_3$) δ 178.4, 158.2, 135.8, 130.5, 127.2, 116.2, 51.6, 48.5, 22.7. LC-MS (ESI) *m*/*z* found: 557 $[M+H]^+$; retention time: 5.56 minutes. HRMS-ESI $[M+H]^+$ calculated for $C_2H_2Br_2N_4O_2S_2^+$: 556.9238, found: 556.9311.

2,2'-methylenebis(4,4-dimethyl-2,4-dihydro-3H-pyrazol-3-one) (11g)

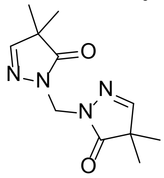

Prepared according to general method II using 100 mg (0.89 mmol) of pyrazolone **31**. The white solid obtained was recrystallized from MeOH to give 74 mg (0.31 mmol, 70%) of the title compound as a white solid. ^{1}H NMR (600 MHz, $CDCl_3$) δ 7.23 (s, 2H), 5.50 (s, 2H), 1.26 (s, 12H). ^{13}C NMR (151 MHz, $CDCl_3$) δ 177.9, 157.1, 51.4, 47.7, 20.5. LC-MS (ESI) *m*/*z* found: 237 $[M+H]^+$; retention time: 2.70 minutes. HRMS-ESI $[M+H]^+$ calculated for $C_{11}H_{17}N_4O_2^+$: 237.1273, found: 237.1346.

1,1'-(ethane-1,2-diyl)bis(3-(3-bromo-4-methoxyphenyl)-4,4-dimethyl-1H-pyrazol-5(4H)-one) (12a)

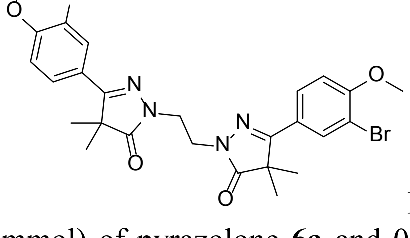

Prepared according to general method IV using 1.0 g (3.37 mmol) of pyrazolone **6a** and 0.145 mL (1.68 mmol) of 1,2-dibromoethane. The crude was purified over SiO_2 using a gradient from 20% EtOAc in n-heptane towards 60% EtOAc to give 0.41 g (0.66 mmol, 39%) of the title compound as a white solid. ^{1}H NMR (600 MHz, $CDCl_3$) δ 7.96 (d, *J* = 2.1 Hz, 2H), 7.62 (dd, *J* = 2.1, 8.6 Hz, 2H), 6.88 (d, *J* = 8.7 Hz, 2H), 4.11 (s, 4H), 3.92 (s, 6H), 1.39 (s, 12H). ^{13}C NMR (151 MHz, $CDCl_3$) δ 178.8, 160.9, 157.3, 131.6, 127.0, 125.3, 112.6, 111.9, 56.7, 48.6, 43.6, 22.7. LC-MS (ESI) *m*/*z* found: 62 $[M+H]^+$; retention time: 5.20 minutes. HRMS-ESI $[M+H]^+$ calculated for $C_{26}H_{29}Br_2N_4O_4^+$: 62.0477, found: 62.0550.

1,1'-(butane-1,4-diyl)bis(3-(3-bromo-4-methoxyphenyl)-4,4-dimethyl-1H-pyrazol-5(4H)-one) (12c)

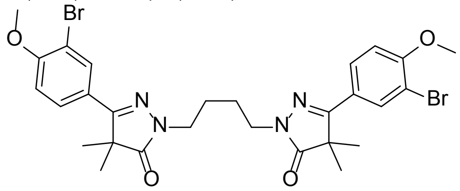

Prepared according to general method IV using 1.0 g (3.37 mmol) of pyrazolone **6a** and 0.20 mL (1.68 mmol) of 1-bromo-4-chlorobutane. The crude was purified over SiO_2 using a gradient from 20% EtOAc in n-heptane towards 60% EtOAc to give 0.6 g (0.93 mmol, 55%) of the title compound as a yellowish solid. ^{1}H NMR (600 MHz, $CDCl_3$) δ 7.95 (d, *J* = 1.9 Hz, 2H), 7.60 (dd, *J* = 1.9, 8.6 Hz, 2H), 6.85 (d, *J* = 8.6 Hz, 2H), 3.92 (s, 6H), 3.79 (s, 4H), 1.78 (s, 4H), 1.46 (s, 12H). ^{13}C NMR (151 MHz, $CDCl_3$) δ 178.6, 160.5, 157.0, 131.1, 126.5, 124.9, 112.3, 111.5, 77.1, 56.4, 48.4, 43.5, 25.2, 22.8. LC-MS (ESI) *m*/*z* found: 647 $[M+H]^+$; retention time: 5.30 minutes. HRMS-ESI $[M+H]^+$ calculated for $C_{28}H_{33}Br_2N_4O_4^+$: 647.0790, found: 647.0863.

2,2'-(pentane-1,5-diyl)bis(5-(3-bromo-4-methoxyphenyl)-4,4-dimethyl-2,4-dihydro-3H-pyrazol-3-one) (12d)

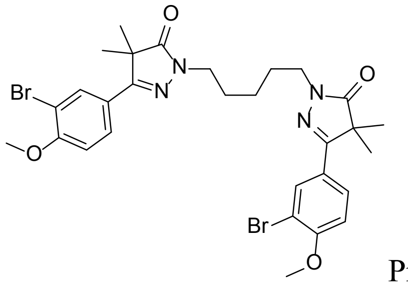

Prepared according to general method IV using 1.0 g (3.37 mmol) of pyrazolone **6a** and 0.23 mL (1.68 mmol) of 1,2-dibromopentane. The crude was purified over SiO_2 using a gradient from 20% EtOAc in n-heptane towards 60% EtOAc to give 0.53 g (0.800 mmol, 47%) of the title compound as a white solid. ^{1}H NMR (600 MHz, $CDCl_3$) δ 8.01 (d, *J* = 2.2 Hz, 2H), 7.67 (dd, *J* = 2.2, 8.6 Hz, 2H), 6.91 (d, *J* = 8.7 Hz, 2H), 3.94 (s, 6H), 3.75 (t, *J* = 7.1, 7.1 Hz, 4H), 1.81 (p, *J* = 7.3, 7.3, 7.3, 7.3 Hz, 4H), 1.44 (s, 12H), 1.39 (p, *J* = 7.5, 7.5, 7.7, 7.7 Hz, 2H). ^{13}C NMR (151 MHz, $CDCl_3$) δ 178.7, 160.8, 157.36, 131.6, 126.9, 125.3, 112.7, 112.0, 77.2, 56.7, 48.7, 44.4, 28.3, 24.1, 23.0. LC-MS (ESI) *m*/*z* found: 661 $[M+H]^+$; retention time: 5.66 minutes. HRMS-ESI $[M+H]^+$ calculated for $C_{29}H_{35}Br_2N_4O_4^+$: 661.0947, found: 661.1020.

References

1. Blaazer, A. R.; Orrling, K. M.; Shanmugham, A.; Jansen, C.; Maes, L.; Edink, E.; Sterk, G. J.; Siderius, M.; England, P.; Bailey, D.; de Esch, I. J. P.; Leurs, R. Fragment-Based Screening in Tandem with Phenotypic Screening Provides Novel Antiparasitic Hits. *Journal of Biomolecular Screening* **2014**.
